# In-silico Investigations of quinine and quinidine as potential Inhibitors of AKR1B1 and AKR1B10: Functional and structural characterization

**DOI:** 10.1371/journal.pone.0271602

**Published:** 2022-10-27

**Authors:** Syeda Abida Ejaz, Amna Saeed, Pervez Rashid Birmani, Khadijah Mohammedsalaeh Katubi, Zainab Mufarreh Elqahtani, M. S. Al-Buriahi, Rabail Ujan, Farhan Siddique, Samia ben Ahmed, Z. A. Alrowaili

**Affiliations:** 1 Department of Pharmaceutical Chemistry, Faculty of Pharmacy, The Islamia University of Bahawalpur, Bahawalpur, Pakistan; 2 Tehsil head quarter hospital, Taunsa Sharif, Pakistan; 3 Department of Chemistry, College of Science, Princess Nourah bint Abdulrahman University, Riyadh, Saudi Arabia; 4 Department of Physics, College of Science, Princess Nourah bint Abdulrahman University, Riyadh, Saudi Arabia; 5 Department of Physics, Sakarya University, Sakarya, Turkey; 6 Dr. M. A. Kazi Institute of Chemistry, University of Sindh, Jamshoro, Pakistan; 7 Laboratory of Organic Electronics, Department of Science and Technology, Linköping University, Norrköping, Sweden; 8 Department of Pharmacy, Royal Institute of Medical Sciences (RIMS), Multan, Pakistan; 9 Departement of Chemistry, College of Sciences, King Khalid University, Abha, Saudi Arabia; 10 Department of Physics, College of Science, Jouf University, Sakaka, Saudi Arabia; Universiti Teknologi Malaysia, MALAYSIA

## Abstract

The aberrant expression of aldo keto reductases (AKR1B1 & AKR1B10) has been extensively studied in different types of cancer especially the colon cancer but a very few studies have yet been reported regarding the discovery of inhibitors for the treatment of colon cancer by targeting these isozymes. Therefore, there is a need of selective inhibitors of both targets for the eradication of colon cancer. Currently, the study is focused on the exploration of two quinolone compounds *i*.*e*., (S)-(6-Methoxyquinolin-4-yl)[(1S,2R,4S,5R)-5-vinylquinuclidin-2-yl]methanol (Quinidine) and (R)-(6-Methoxyquinolin-4-yl)[(1S,2S,4S,5R)-5-vinylquinuclidin-2-yl]methanol (Quinine) as the potential inhibitors of AKR1B1 and AKR1B10 via detailed in-silico approach. The structural properties including vibrational frequencies, dipole moment, polarizability and the optimization energies were estimated using density functional theory (DFT) calculations; where both compounds were found chemically reactive. After that, the optimized structures were used for the molecular docking studies and here quinidine was found more selective towards AKR1B1 and quinine exhibited maximum inhibition of AKR1B10. The results of molecular docking studies were validated by molecular dynamics simulations which provided the deep insight of stability of protein ligand complex. At the end, the ADMET properties were determined to demonstrate the druglikeness properties of both selected compounds. These findings suggested further exploration of both compounds at molecular level using different in-vivo and in-vitro approaches that will lead to the designing of potential inhibitor of AKR1B1/AKR1B10 for curing colon cancer and related malignancies.

## Introduction

Aldo-keto reductases: AKRs are found in nearly all phyla and are primarily monomeric soluble proteins (34–37 kDa) that function as NAD(P)(H) dependent oxidoreductases [1]. Up to date, huge genome data sets has been reported via *in-silico* approaches and it has been reported that this protein superfamily has 190 identified proteins which are further divided into 16 families [2]. From these sixteen families, the total fifteen members of AKR family have been identified in human which include: AKR1A, AKR1B, AKR1C, AKR1E, AKR6A and AKR7A subfamilies. Among these sub-families, AKR1B has gained a lot of attention because of its extensive role in different types of cancer. The AKR1B subfamily comprises of three isozymes *i*.*e*., AKR1B1 (aldose reductase), AKR1B10 (aldose reductase-like protein-1) and AKR1B15 [3, 4] and here we are focused on the exploration of inhibitors of AKR1B1 and AKR1B10. Both are expressed in various organs but majorly in breast, liver, lungs, pancreas and small intestine [5] and share 71% amino acid sequence similarity. Both targets are extensively involved in different types of cancer, especially the colon cancer and lung cancer, where their level is found to be overexpressed [6]. With respect to the other roles of these targeted AKRs, AKR1B10 is involved in maintaining the retinoic acid homeostasis, which is considered as most important factor that promote the cell differentiation [7]. Moreover, AKR1B10 affects the growth of cells and survival through affecting lipid production and membrane function by blocking the ubiquitin-dependent degradation of acetyl-CoA carboxylase [8].

Another isozyme *i*.*e*., maintaining the homeostasis of reactive oxygen species (ROS) is the responsibility of AKR1B1 and thus crucial for the regulation of inflammatory transcription factors (TFs). Aberrant expression of AKR1B1 resulted in the activation of various signaling pathways such as NFκB, an ubiquitous transcription factor found in various cancer [9]. On the basis of these facts, it can be observed that both AKR1B10 and AKR1B1 are aberrantly expressed in different cancer so there is a need to explore potential inhibitors of both targets [10].

**Scheme 1:**
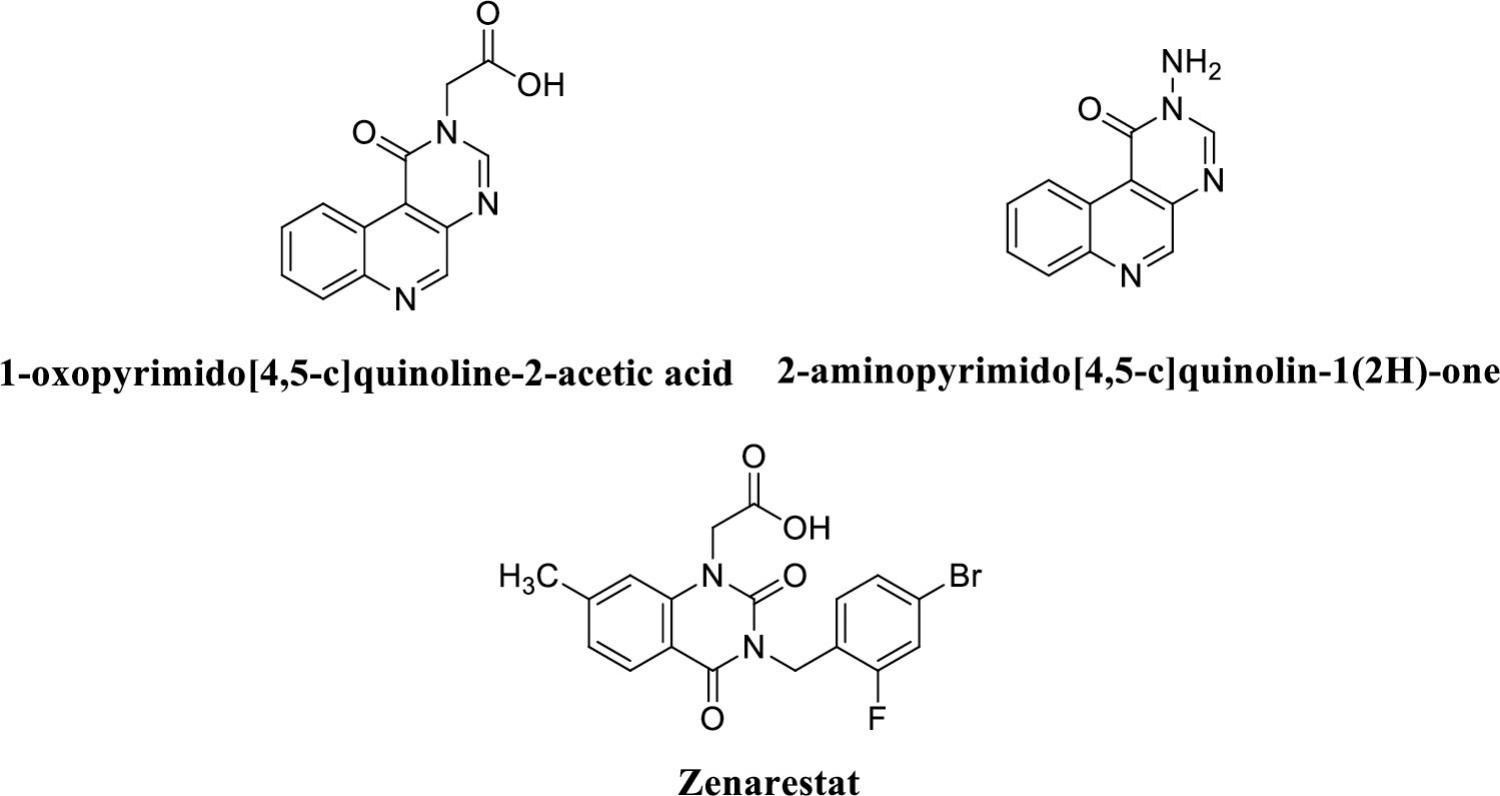
Already reported aldo-keto reductase inhibitors [14, 15].

Among the different type of heterocyclic compounds, quinolone derivatives have been reported as the significant class of compounds with varying biological activities including analgesics, local anesthetic, anti-inflammatory, anti-cancer, pain relief, neuropharmacological, anti-microbial, anti-fungal and also the anti-malarial activities [11–13]. Due to their remarkable chemical and physical properties, quinolone derivatives have been the subject of several structural and theoretical investigations [4]. Many studies have been reported on the quinolone alkaloids as the anticancer agents but very few of them have been reported as the inhibitor of aldo-keto reductases. Scheme 1 shows the already reported aldo-keto reductase inhibitors; 1-oxopyrimido[4,5-c]quinoline-2-acetic acid (IC_50_ value = <1 mM), 2-aminopyrimido[4,5-c]quinolin-1(2H)-one (IC_50_ value = <1 mM) and zenarestat exhibited the IC_50_ value of 44 nM [14, 15].

Here only two quinolones: quinine and quinidine were selected and the study’s objective was to investigate the reactivity and stability of the quinolone derivatives comprehensively as the potential inhibitors of AKR1B1 and AKR1B10. For this, the optimized geometry analysis has been used to interpret the structural information for the compounds where the dipole moment, polarizability and optimization energy of the selected compounds were calculated. Further, molecular docking studies were performed using geometrically optimized compounds against the targets AKR1B1 and AKR1B10 and the results were supported by molecular dynamic simulations. The physicochemical parameters (ADMET properties) were estimated for considering the druglikeness properties of both compounds. The results of the current study are very promising therefore suggested that both compounds can be used in future for the synthesis of more potential inhibitors of the selected targets, for the treatment of respective cancers caused due to over-expression of AKR1B1 and AKR1B10.

## Experimental

### Density functional theory (DFT)

The Gaussian 09 package (Rev.E.01) [16] with default settings was used for all calculations with B3LYP functional in SVP basis set [17]. Calculating the electronic structure of atoms and molecules is effectively done using this theory. The following information will be determined by the current study *i*.*e*., optimized geometric parameters, the frontier molecular orbital (FMO), global and local reactivity descriptors and molecular electrostatic potential (MEP) [18]. Check files were viewed using Guass View 6 [19].

### Molecular docking

#### Preparation of ligand

The selected ligands were obtained from PubChem [20] and their energy minimization was carried out by using chem3D pro 12 [21]. The structures were then saved into desired formats (pdb/ sdf) that were used for further studies.

#### Preparation of protein

The protein targets AKR1B1, AKR1B10, NF-κB, caspase-3 and cellular tumor antigen P53 were taken from RCSB (PDB) protein data bank with the PDB ID: 6F7R, 4GQG, 1NFI, 2C1E and 4BUZ, respectively [22]. Targeted protein was prepared in both AutoDock [23] and Molecular Operating Environment (MOE) [24], by removing hetatoms and water molecules. Only polar hydrogens were integrated while utilizing both softwares. AutoDock was used to add Kollman charges. MOE was used to perform partial charges and 3D protonation.

#### Docking protocol

The most important parameter while using AutoDock was setting grid box for active site. It was prepared by using 60 × 60 × 60 dimensions of x, y and z axis, respectively with gap of 0.375 Å and population size was set to 150 with 100 no. of runs. Lamarckian genetic algorithm (LGA) was adopted for docking [25].

In MOE, dummies were created on the amino acids of active pocket. MMFF94x forcefield was loaded to estimate the forces between the atoms [26]. Triangle Matcher algorithm was chosen for docking. Runs were set up to 100. The results of Autodock were visualized in Discovery studio visualizer (version 2020) [27] while the results of MOE were visualized in the window of MOE [28].

#### Validation

Validation was being processed by calculating RMSD value. The conformation whose RMSD value is ≤ 2 would be considered as the best pose [29]. No other ways for validation are available [30]. Furthermore, co-crystal ligand (NAP) docking was used to evaluate and verify our findings.

### Molecular dynamic simulation

For 100 nanoseconds, MD simulations were carried out for both the complexes by using the same protocol as reported earlier [31]. Protein and protein-ligand complexes were prepared and then NPT equilibration were carried out as discussed previously [31]. The complete protocol of MD simulation is given in the supplementary file.

### ADMET properties

The different physicochemical properties i.e., absorption, distribution, metabolism, excretion and toxicity are collectively called ADMET properties that are calculated in order to find a compound’s drug-likeness. These all mentioned properties were analyzed as mentioned in our previous article [32]. By the assistance of web server i.e., ADMET lab 2.0, the ADMET characteristics of the quinolone derivatives were estimated [33]. The detailed information about the ADMET properties is given in the supplementary data.

## Results and discussion

### Density functional theory (DFT)

#### Molecular geometry

**Fig 1:**
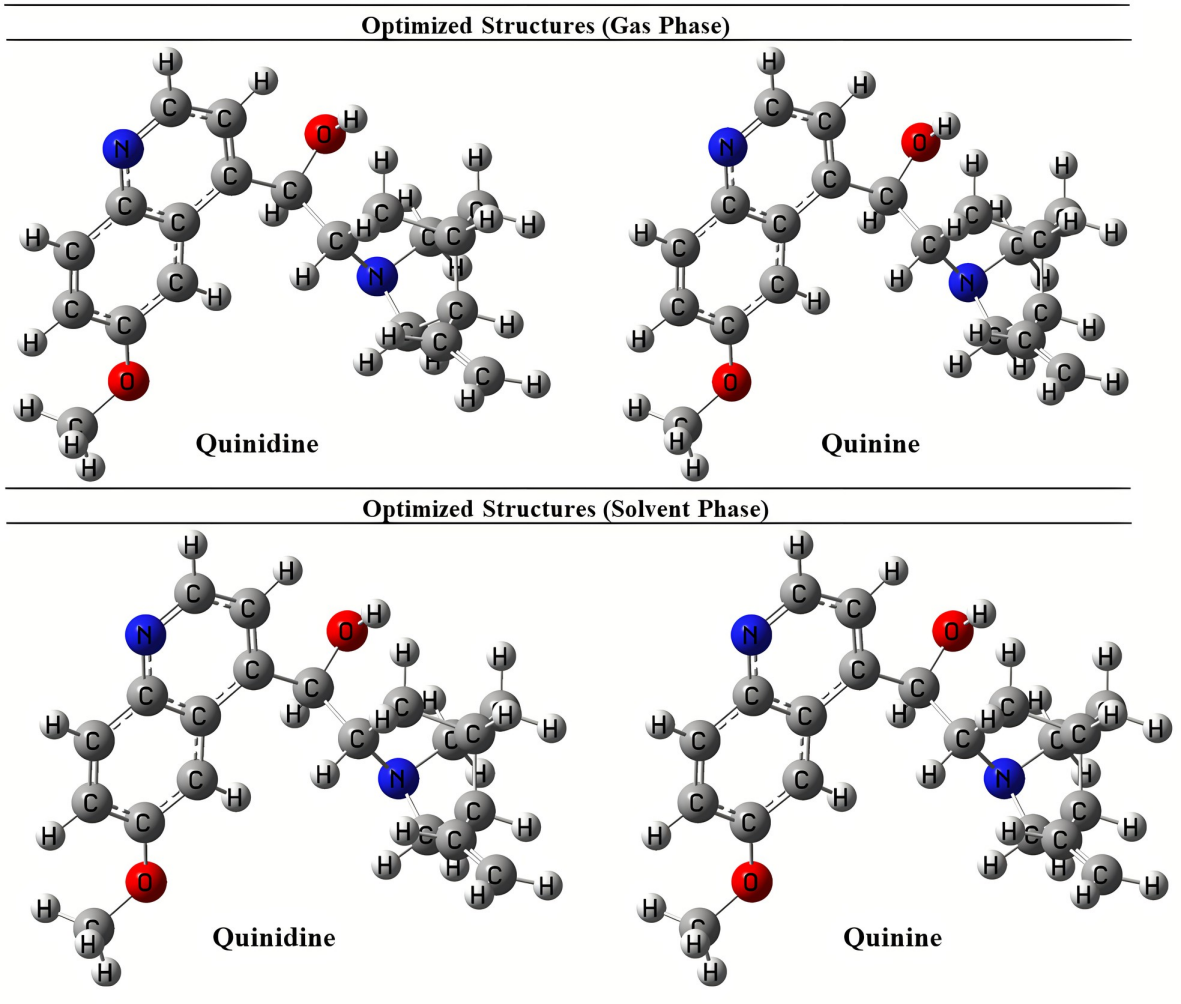
Optimized structures of quinolone derivatives.

The optimized geometry of quinolone alkaloids which was performed by B3LYP functional methods with basis set of SVP are depicted in Fig 1.

The optimized energies along with their dipole moments and polarizability, obtained by the DFT/B3LYP/SVP method are listed in Table 1.

**Table 1:** Calculation of energetic parameters and quantum chemical descriptors of quinolone derivatives.

Compound	Gas phase			Solvent Phase (water)		
	Optimization energy (hartree)	Polarizability (α) (a.u.)	Dipole moment (Debye)	Optimization energy (hartree)	Polarizability (α) (a.u.)	Dipole moment (Debye)
Quinidine	-1035.784	236.1147	2.4307	-1035.796	318.855	3.054
Quinine	-1035.7845	236.1147	2.4307	-1035.7962	318.8558	3.054

With reference to bond lengths and bond angles, it was discovered that the geometric structure has an impact on the electrical properties. Using DFT/B3LYP/SVP models, the most stable optimal structural parameters like bond length, bond angle, and dihedral angles were discovered.

The DFTs were used for both optimization in gas phase and solvent phase. The molecular geometry and molecular descriptors were used for the calculation of reactivity, shape and binding properties of the selected molecules.

In addition to this, the dipole moment is a global measure of the accuracy with which the electron density of a polar molecule is computed. Moreover, the dipole moments influence the molecule’s interactions with other molecules and electric fields. It allows the identification and enumeration of intermolecular interactions. Here both compounds showed maximum dipole moments and formed strong bonding and non-bonding interactions with the targeted proteins. In addition, to evaluate the accuracy of a quantum chemical approach, the links between the electronic structures of molecules were established through the electric properties of molecules.

#### Frontier molecular orbital (FMOs)

The many types of reactions are described and the most reactive position in conjugated systems is understood through the study of molecular orbitals and their energies. Information about a molecule’s biological and chemical activity can be found in the energies of the highest occupied molecular orbital (HOMO), lowest unoccupied molecular orbital (LUMO), and their energy gap. A narrow orbital gap indicated a highly polarizable molecule, which is typically associated with strong chemical reactivity and low kinetic stability. Currently, **Quinidine** and **Quinine** are highly polarized and chemically reactive in solvent phase as compared to gas phase. The HOMO, or outer orbital containing electrons, acts as an electron donor, and hence the ionization potential (I) is proportional to the HOMO’s energy. On either hand, LUMO is capable of accepting electrons, and its electron affinity (A) is proportional to its energy [34].

**Fig 2:**
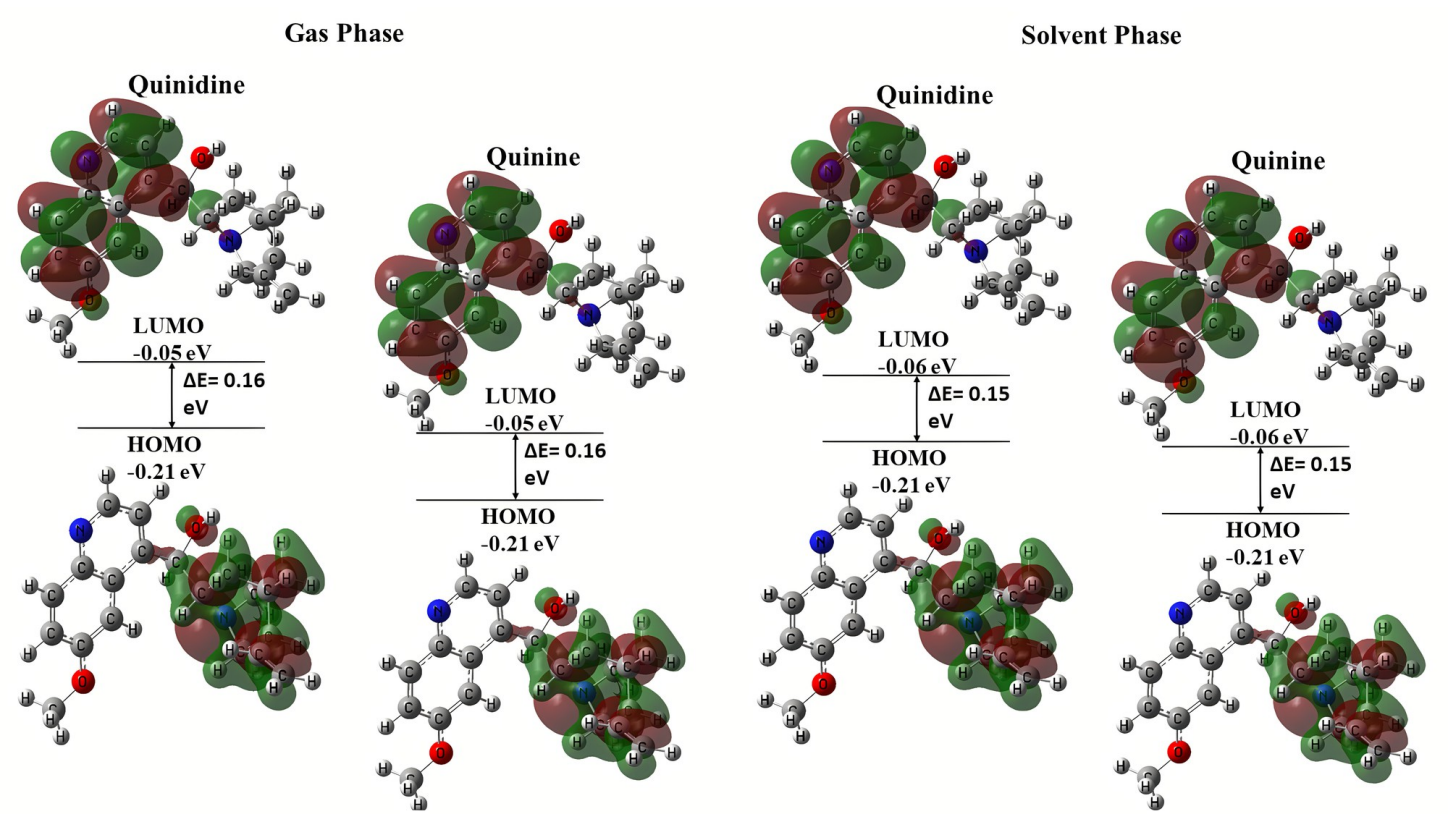
HOMO and LUMO structures of quinolone derivatives in gas and solvent (water) phase.

In gas phase, both the compounds **Quinidine** and **Quinine** had shown the same energy gap of 0.16 eV. On contrast in solvent phase (water), both the compounds had shown the same energy gap of 0.15 eV. They might have shown the same energy gaps because they are optical isomers of each other. HOMO-LUMO structures of compounds **Quinidine** and **Quinine** in the gas phase and solvent phase (water) are depicted in Fig 2.

#### Global chemical reactivity descriptors

By using energy values of HOMO LUMO, we evaluated the following parameters mentioned in Table 2 by using following equations:

Hardness: η = 1/2(ELUMO—EHOMO); Softness: S = 1/2η; Electronegativity: χ = -1/2(ELUMO + EHOMO); Chemical potential: μ = —χ; Electrophilicity index: ω = μ/2η

**Table 2:** Quantum chemical descriptors of quinolone derivatives in gas phase.

Gas phase							
Compound	Hardness (η)		Softness (S)	Electronegativity (X)	Chemical Potential (μ)		Electrophilicity Index (ω)
Quinidine	0.078		6.405	0.135	-0.135		0.118
Quinine	0.078		6.405	0.135	-0.135		0.118
**Compound**	**Electrodonating power (ω** ^**-**^ **)**			**Electroaccepting power (ω** ^**+**^ **)**			**Net Electrophilicity (Δω** ^**±**^ **)**
Quinidine	0.195			0.059			0.254
Quinine	0.195			0.059			0.254
**Solvent phase (water)**							
**Compound**	**Hardness (η)**	**Softness (S)**		**Electronegativity (X)**		**Chemical Potential (μ)**	**Electrophilicity Index (ω)**
Quinidine	0.076	6.603		0.140		-0.140	0.129
Quinine	0.076	6.603		0.140		-0.140	0.129
**Compound**	**Electrodonating power (ω** ^**-**^ **)**			**Electroaccepting power (ω** ^**+**^ **)**			**Net Electrophilicity** **(Δω** ^**±**^ **)**
Quinidine	0.208			0.069			0.277
Quinine	0.208			0.069			0.277

The Compounds with the lowest energy gap are softest, while those with bigger gaps are hard. Here, in the case with **Quinidine** and **Quinine,** both showed the same significant values as they are the optical isomers of each other. Thus, both the compounds behave softer in solvent phase as compared to gaseous phase. The highest HOMO energy of a chemical explains why it is one of the strongest electron donors. Compounds in solvent phase with the lowest anticipated LUMO energy are the most effective electron acceptors. The HOMO and LUMO electron orbital energies are related to two parameters: ionization potential (I) and electron affinity (A). The chemical reactivity of substances varies according to their structures. On the basis of these facts it was observed that the compounds were found more reactive in solvent phase than in gas solvent due to their net electrophilicity, which is 0.277 in solvent phase.

#### Molecular electrostatic potential

**Fig 3:**
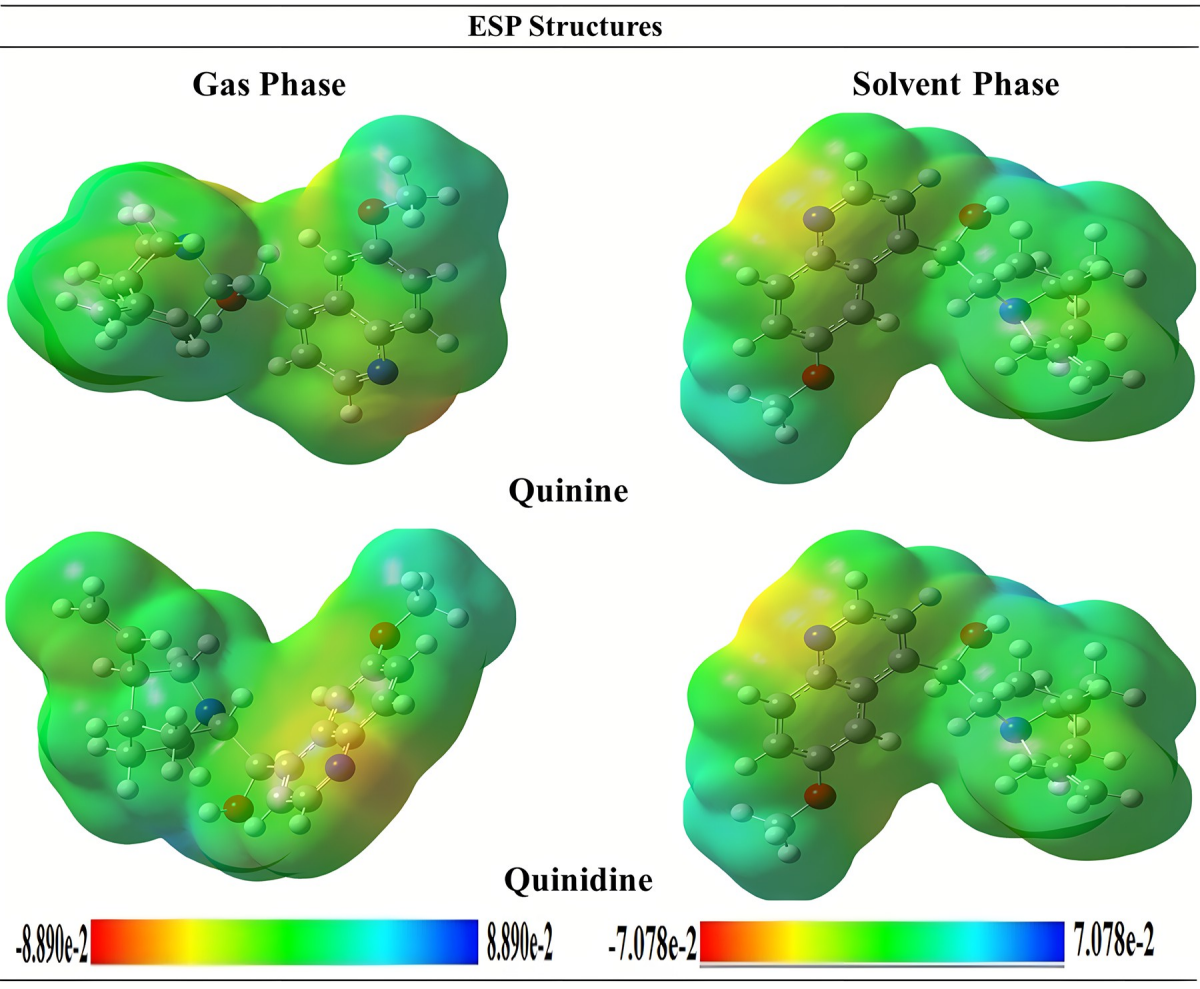
Molecular electrostatic potential (MEP) structures of quinolone derivatives in gas phase and solvent phase as well.

The MEP (molecular electrostatic potential) analysis is used to understand the electrophilic and nucleophilic attacking sites of the molecules. To evaluate the distribution of electrical charge, electrostatic potential is frequently used. It is an essential tool to observe specific biological processes and hydrogen bonding interactions as well as electrophilic and nucleophilic attacks on molecules. An understanding of how various geometries interact can be gained from the surface and contours. The electron density and electrostatic potential of the aforementioned compounds are shown in Fig 3. The graphic showed that the electron density is equally dispersed throughout the molecules, according to ESP bar data, negative ESP is only localized to particular regions of a molecule. Because ESP and a molecule’s electronegativity and partial charges are related, this inference is evident. Different colours displayed in the ESP bar represented different electrostatic potential values. Red represents a region of high electrostatic potential, blue a region of high electrostatic potential, and green a zone of zero electrostatic potential [34].

The very negative potential is clearly restricted to the nitrogen and oxygen atoms of the pyrazole ring, as evidenced by this observation. These discoveries are significant for illustrating how electrophilic and nucleophilic forces act upon molecules.

### Molecular docking

#### Molecular docking with AKR1B1

**Fig 4:**
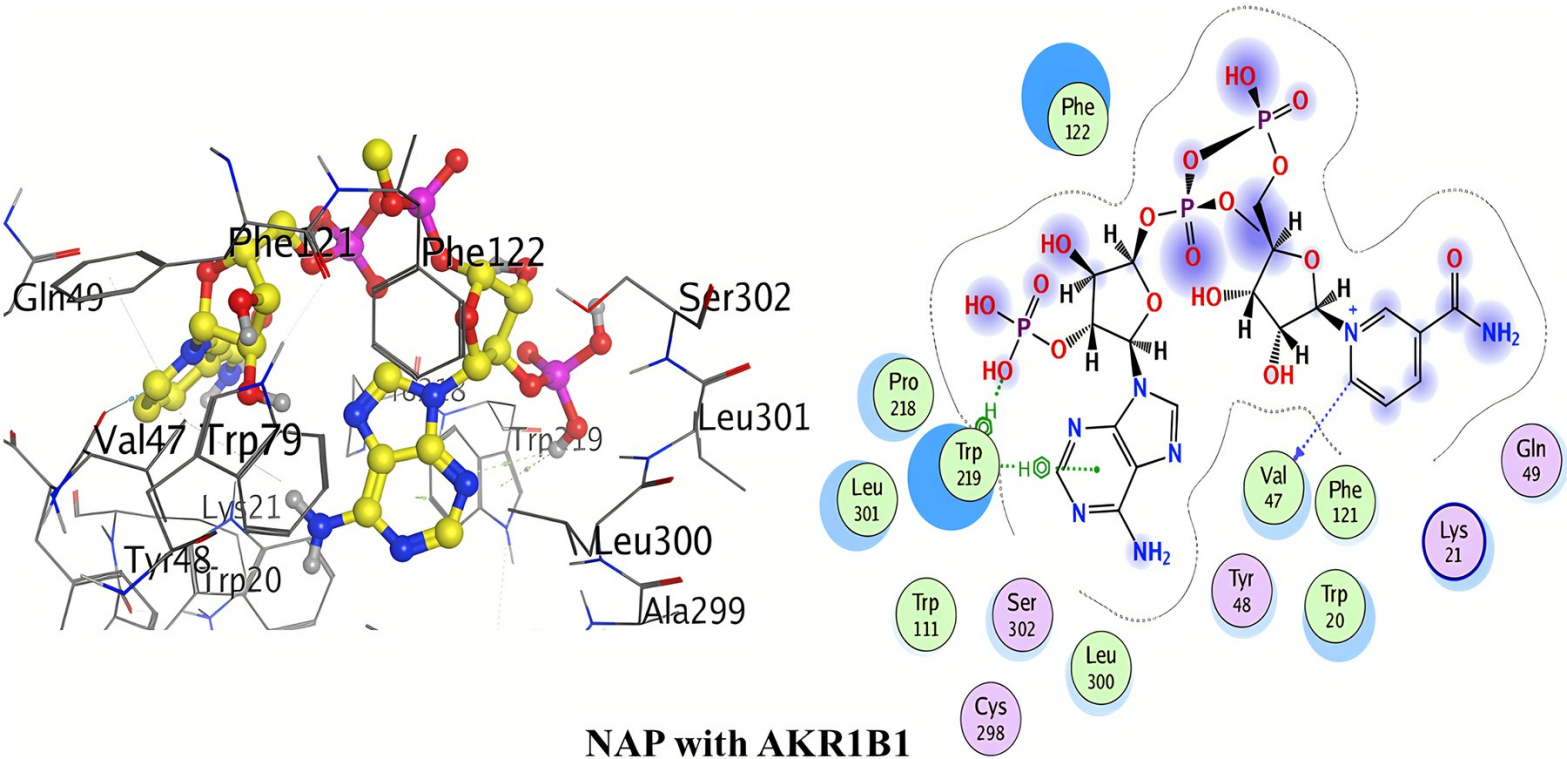
3D and 2D interactions of Nap (co-crystal ligand) with active site of AKR1B1.

The detailed 3D and 2D binding interactions of AKR1B1 with NAP (co-crystal ligand) is shown below in Fig 4. Hydrogen bond complex was observed by oxygen of trimethyl phosphate, oxygen of benzamide, hydroxyl group of dimethyl tetrahydrofuran-3, 4-diol of NAP with amino acid residue Lys262. One of the hydrogen bond interactions was formed between oxygen of methyl dihydrogen phosphate of NAP and Lys21. Oxygen of trimethyl tetrahydrofuran-3-ol of NAP exhibited hydrogen bonding with Arg268. Hydrogen bond interaction was also formed between hydroxyl group of dimethyl tetrahydrofuran-3, 4-diol of NAP and Arg217. Strong hydrophobic interaction (Pi-sigma) was seen between Pyrimidin-4-amine group of NAP and Leu228. Electrostatic interaction (Pi-cation) was observed by Phosphorous of methyl dihydrogen phosphate of NAP with Asp216. Detailed binding energies of quinolone derivatives with both targets AKR1B1 and AKR1B10 are shown in Table 3.

**Table 3:** Binding energies of quinolone derivatives with target AKR1B1 and AKR1B10 (kJ/mol).

	Code	AKR1B1		AKR1B10	
		MOE	AutoDock	MOE	AutoDock
**1.**	**Quinine**	-29.04	-28.56	-31.02	-35.84
**2.**	**Quinidine**	-31.08	-38.55	-29.2	-31.04
**Co-crystal**	**NAP**	-29.48	-22.92	-27.44	-18.16

**Fig 5:**
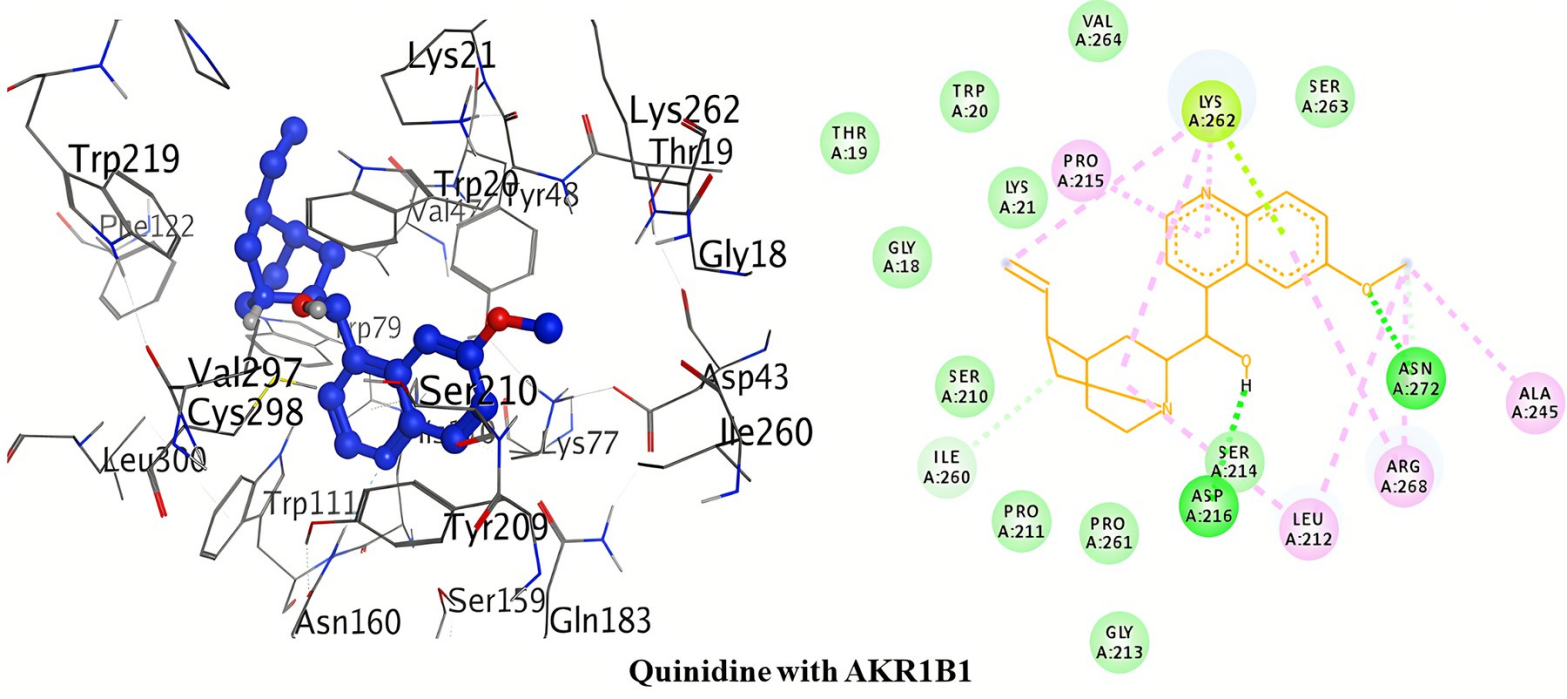
3D and 2D interactions of quinidine with active site of AKR1B1.

The detailed 3D and 2D binding interactions of AKR1B1 with Quinidine is shown below in Fig 5. The amino acid residues of active site which were involved in hydrogen bonding with quinidine included; Asn272 and Asn216. The hydrogen bonding is of significant importance in protein-ligand interaction to check the inhibitory action. One of the hydrogen bond was created by oxygen of 6-methoxyquinoline of quinidine with Asn272. Other hydrogen bond was formed by hydrogen of 1-(quinolin-4-yl)ethanol of quinidine with Asn216. No other strong Pi-Pi and Pi-cation interactions were observed.

**Fig 6:**
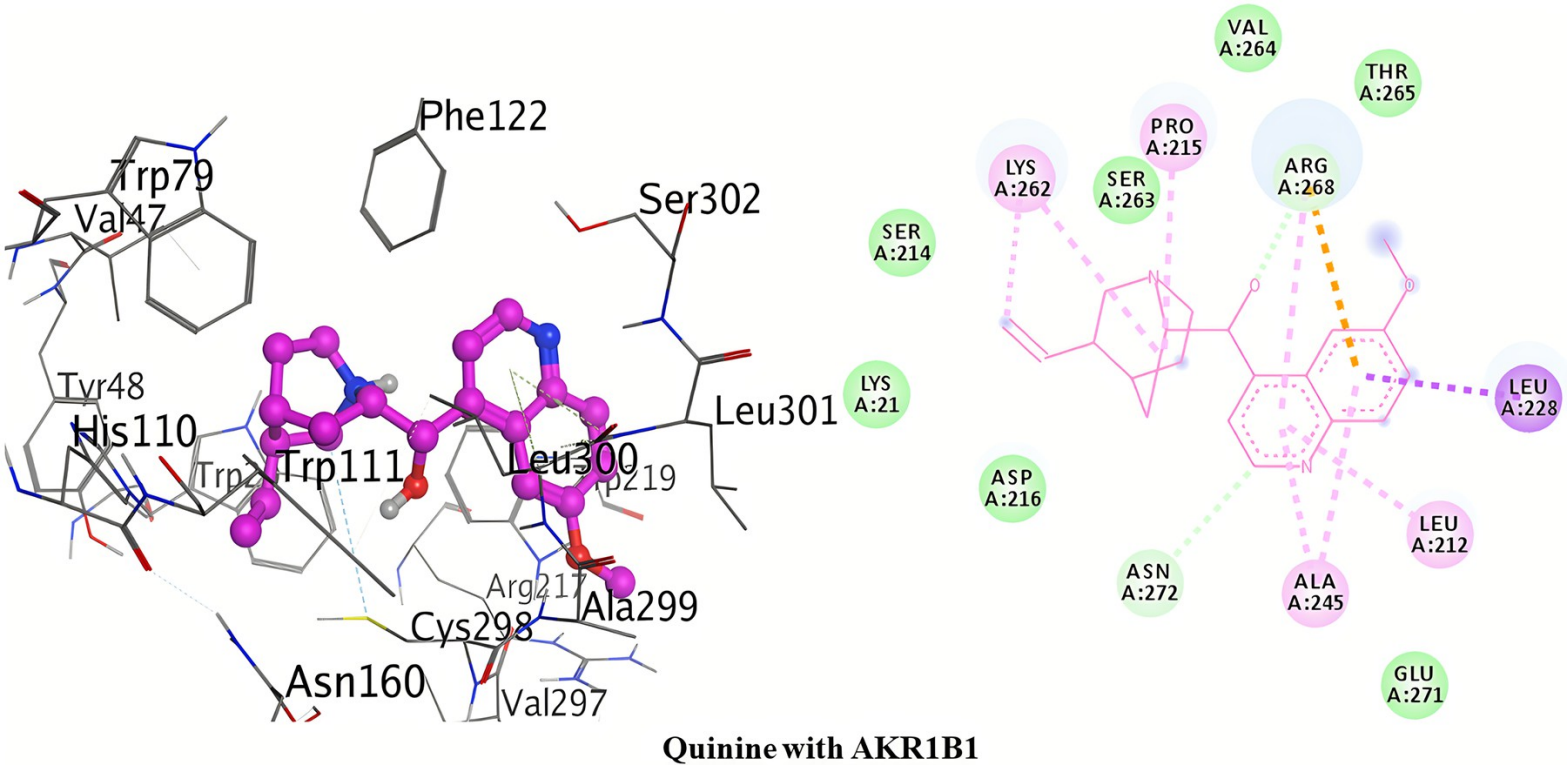
3D and 2D interactions of quinine with active site of AKR1B1.

The detailed 3D and 2D binding interactions of AKR1B1 with Quinine is shown in Fig 6. After analyzing the binding interactions, two carbon hydrogen bonds and one Pi-cation interaction were found. The amino acid residues of active site which were involved in carbon hydrogen bond with quinine included; Asn272 and Arg268. One carbon hydrogen bond was formed by pyridine ring of quinidine with Asn272. One carbon hydrogen bond was formed by oxygen of 6-methoxyquinoline with Arg268. One Pi-cation interaction was formed by anisole ring of 6-methoxyquinoline with Arg268. No other strong Pi-Pi interactions were observed.

#### Molecular docking with AKR1B10

**Fig 7:**
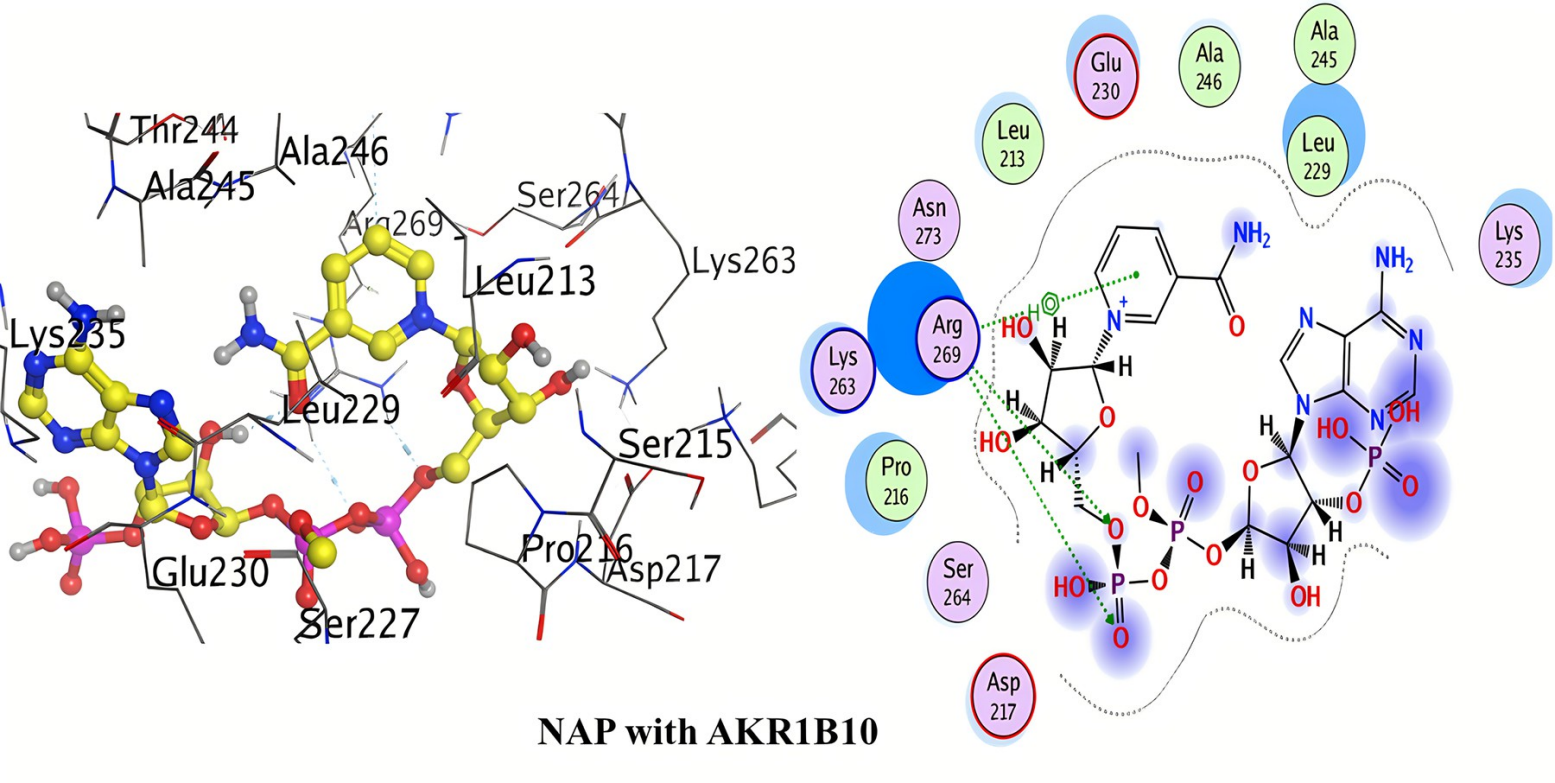
3D and 2D interactions of Nap (co-crystal ligand) with active site of AKR1B10.

The detailed 2D and 3D binding interactions of AKR1B10 with NAP (co-crystal ligand) is shown above in Fig 7. Hydrogen bond complex was observed between hydrogen of pyrimidine-4-amine of NAP and Pro216. Hydrogen bonding was also observed between oxygen of acetamide of NAP and Lys22. Another hydrogen bond complex was formed by hydroxyl group of dimethyl hydrogen phosphate of NAP with Val265.

**Fig 8:**
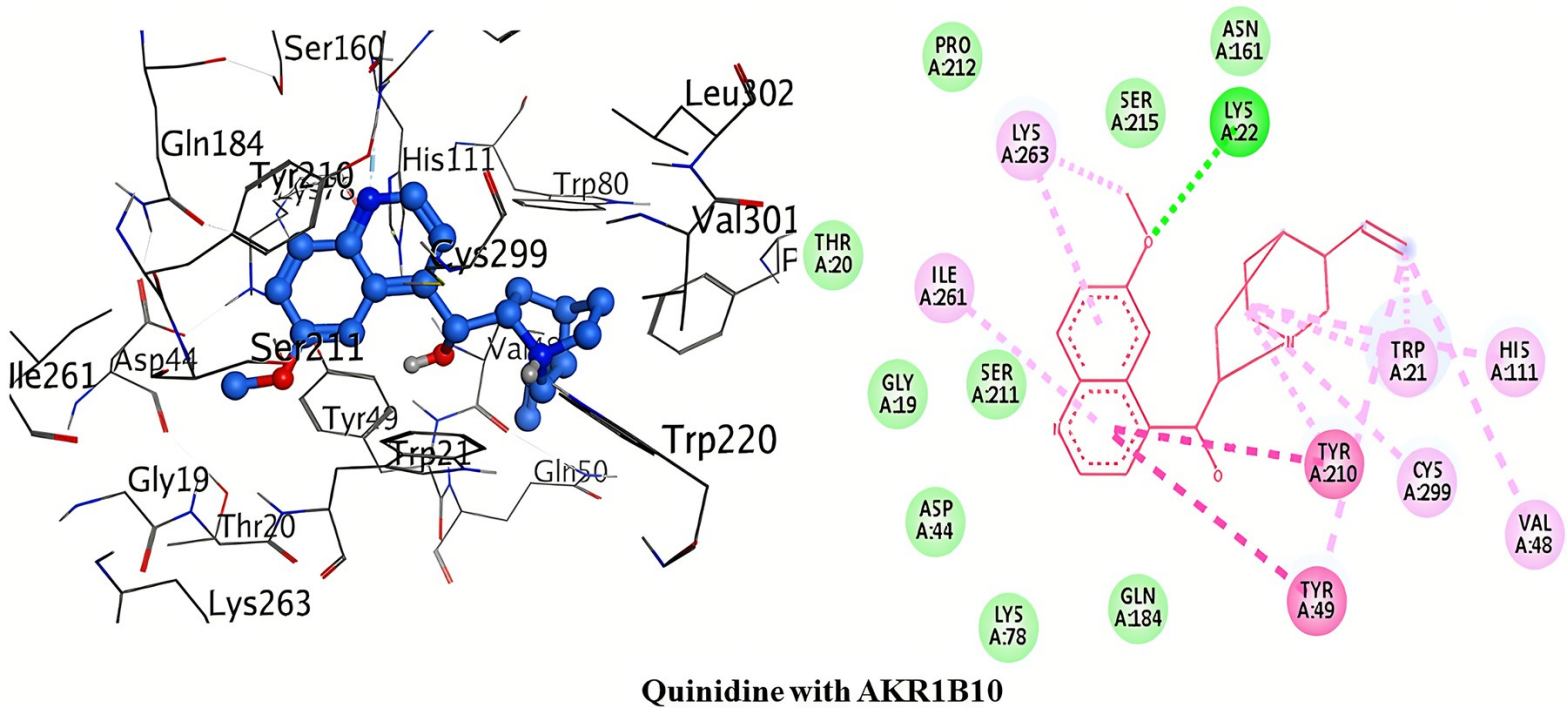
3D and 2D interactions of quinidine with active site of AKR1B10.

The detailed 3D and 2D binding interactions of AKR1B10 with Quinidine is shown in Fig 8. The amino acid residue of active site which was involved in hydrogen bonding with quinidine included; Lys22. This hydrogen bonding is of chief importance in protein-ligand interaction. Observing the binding interactions predicted only one hydrogen bond. This one hydrogen bond was formed by oxygen 6-methoxyquinoline of quinidine with Lys22. Pi-Pi stacked/T-shaped interaction was formed by Tyr210 and Tyr49 amino acids with 6-methoxyquinoline of quinidine. No Pi-cation interaction was shown with quinidine.

**Fig 9:**
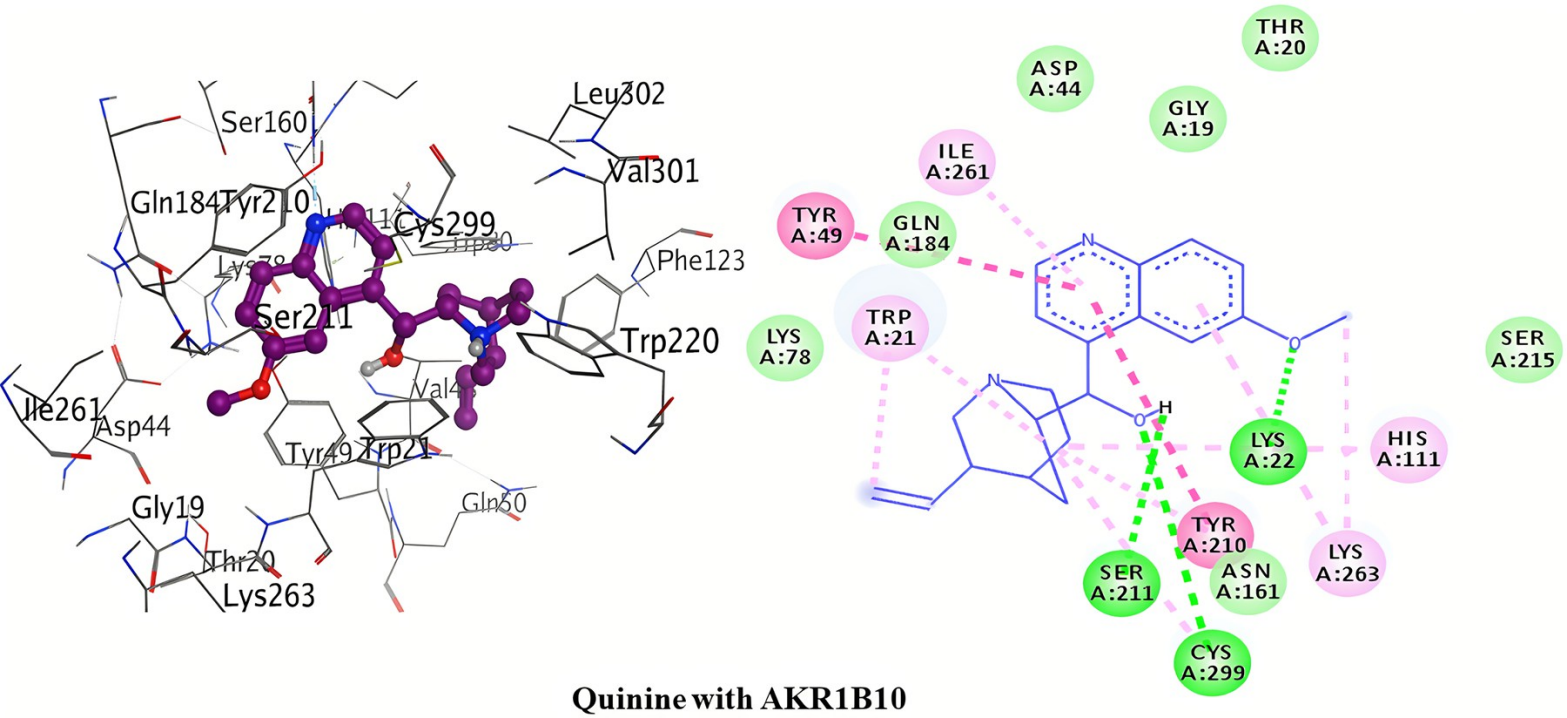
3D and 2D interactions of quinine with active site of AKR1B10.

The detailed 3D and 2D binding interactions of AKR1B10 with Quinine is shown in Fig 9. The amino acid residues of active site which were involved in hydrogen bonding with quinine included; Lys22, Ser211 and Cys299. This hydrogen bonding is of chief importance in protein-ligand interaction. Analyzing the binding interactions showed three hydrogen bonds. One hydrogen bond was formed by oxygen of 6-methoxyquinoline of quinine with Lys22. Ser211 amino acid formed hydrogen bond with hydrogen of 1-(quinoline-4yl)ethanol of quinine. The third hydrogen bond formed between Cys299 and oxygen of 1-(quinoline-4yl)ethanol of quinine. Pi-Pi stacked/T-shaped interaction was formed by Tyr210 and Tyr49 amino acids with 2,3-dihydropyridine of quinine. No Pi-cation interaction was shown with quinine.

Conclusively, the above detailed results of molecular docking has been shown that binding affinities for the selected compounds (quinine and quinidine) are better (lowest) than that of the co-crystal ligand (NAP). Moreover, scattering of docking scores was seen when repeated docking runs were performed. Under the identical docking techniques, the docking programme provided consistently variable docking results. Massive and bulkier binding sites typically exhibit a high degree of dispersal in terms of docking poses and scores, as there are more conformational positions for the ligand. Thus, AutoDock was determined to be the better software of the two due to its lower dispersion when compared to MOE over repeated docking protocols.

#### Structure activity relationship of quinolone derivatives

The structure activity relationship of these quinolone derivatives (quinine and quinidine) was studied on the basis of hydrogen bond interactions observed during molecular docking. Against **AKR1B1**, quinidine was found to be the most potent inhibitor with two hydrogen bonds formation (binding energy = -38.55 kJ/mol). One of the hydrogen bond was formed due to the methoxy substitution at position-6 of quinoline ring of quinidine. Another hydrogen bond formation was observed by ethanol substitution at position-4 of quinoline ring of quinidine. In case of AKR1B1, quinine didn’t form conventional hydrogen bond within the active pocket’s amino acid residues of AKR1B1.

Against **AKR1B10**, quinine was found to be the most potent inhibitor with three hydrogen bonds formation (binding energy = -35.84 kJ/mol). One of the hydrogen bond was formed due to the methoxy substitution at position-6 of quinoline ring of quinine. Other two hydrogen bonds formation were observed by ethanol substitution at position-4 of quinoline ring of quinine.

However, in case of quinidine one hydrogen bond was formed due to the methoxy substitution at position-6 of quinoline ring within the active pocket’s amino acid residues of AKR1B10.

#### Molecular docking with nuclear factor kappa B (NF-κB)

In order to authenticate the anti-cancer potential of quinolone alkaloids, molecular docking is expanded with following targets *i*.*e*., nuclear factor kappa B (NF-κB), cellular tumor antigen P35 and caspase-3.

**Fig 10:**
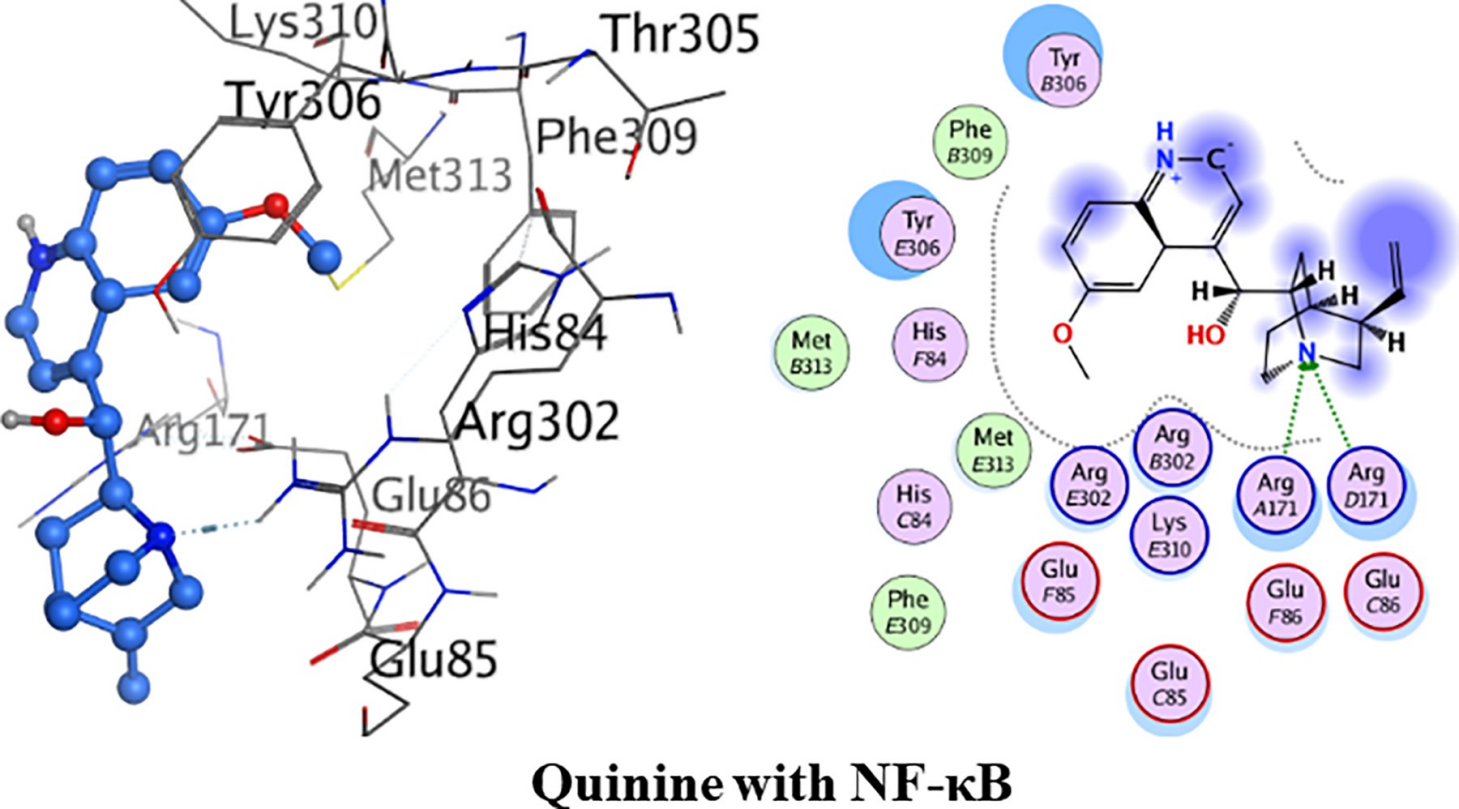
3D and 2D interactions of quinine with active site of nuclear factor kappa B (NF-κB).

The detailed 3D and 2D interactions are shown in Fig 10 below. The amino acid residues of the active pocket of NF-κB are; Arg302, Lys310, Tyr306, His84, Met313, Arg171, Glu86, Ala88 and Lys87. Quinine had shown the minimum binding energy of -25.08 kJ/. The amino acid residue Arg171 was observed to form one hydrogen bond interaction with amine group of quinine whileMet313 and Phe309 were involved in van der Waals interactions.

**Fig 11:**
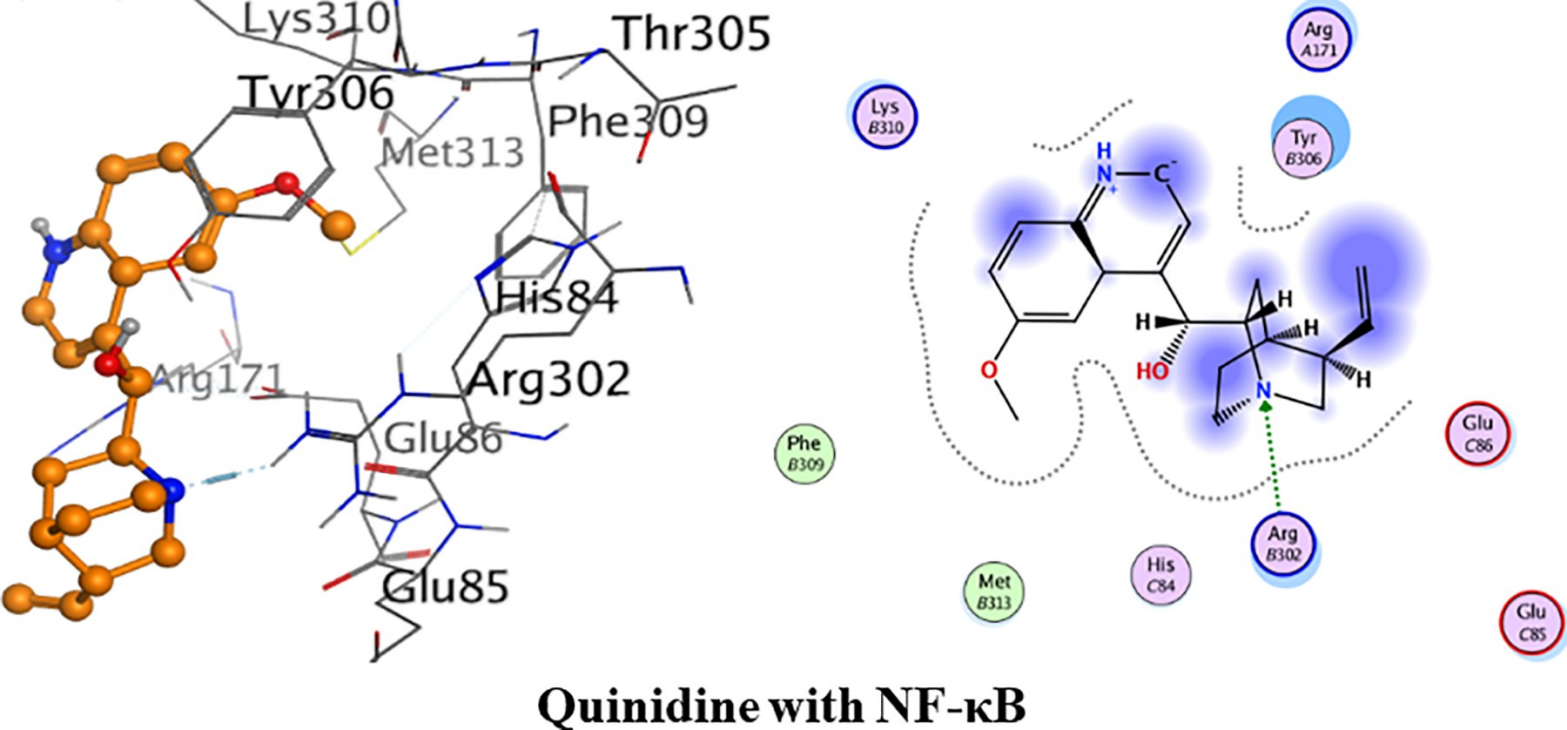
3D and 2D interactions of quinidine with active site of nuclear factor kappa B (NF-κB).

Detailed binding interactions of quinidine with NF-κB are shown in Fig 11 below. Quinidine had shown the binding energy of -23.60 kJ/mol. One hydrogen bond interaction was formed between amino acid residue Arg302 and amine group of quinidine. Met313 and Phe309 were involved in van der Waals interaction.

#### Molecular docking with cellular tumor antigen P53

**Fig 12:**
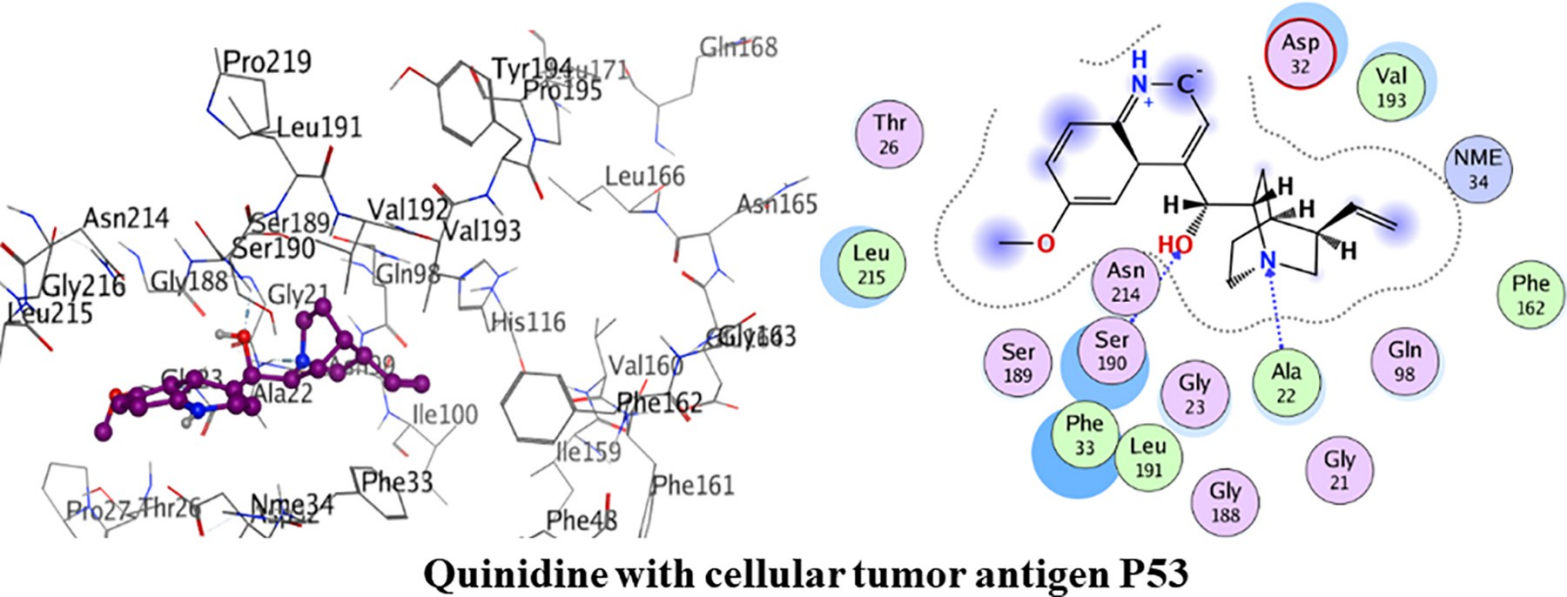
. 3D and 2D interactions of quinidine with active site of cellular tumor antigen P53.

The amino acid residues of the active pocket of cellular tumor antigen P53 are; Leu215, Asp231, Thr26, Pro27, Gly188, Val232, Asp32 and Asn214. Detailed binding interactions of quinidine with cellular tumor antigen P53 are shown in Fig 12 below. Quinidine had shown the minimum binding energy of -20.68 kJ/mol and interacted with two hydrogen bond. Amino acid residue Ala22 was observed in formation of hydrogen bond interaction with amine group of quinidine. Another hydrogen bond interaction was formed between amino acid residue Ser190 and hydroxyl group of quinidine. Phe162, Val193, Leu191, Leu215 and Phe33 were involved in van der Waals interaction.

**Fig 13:**
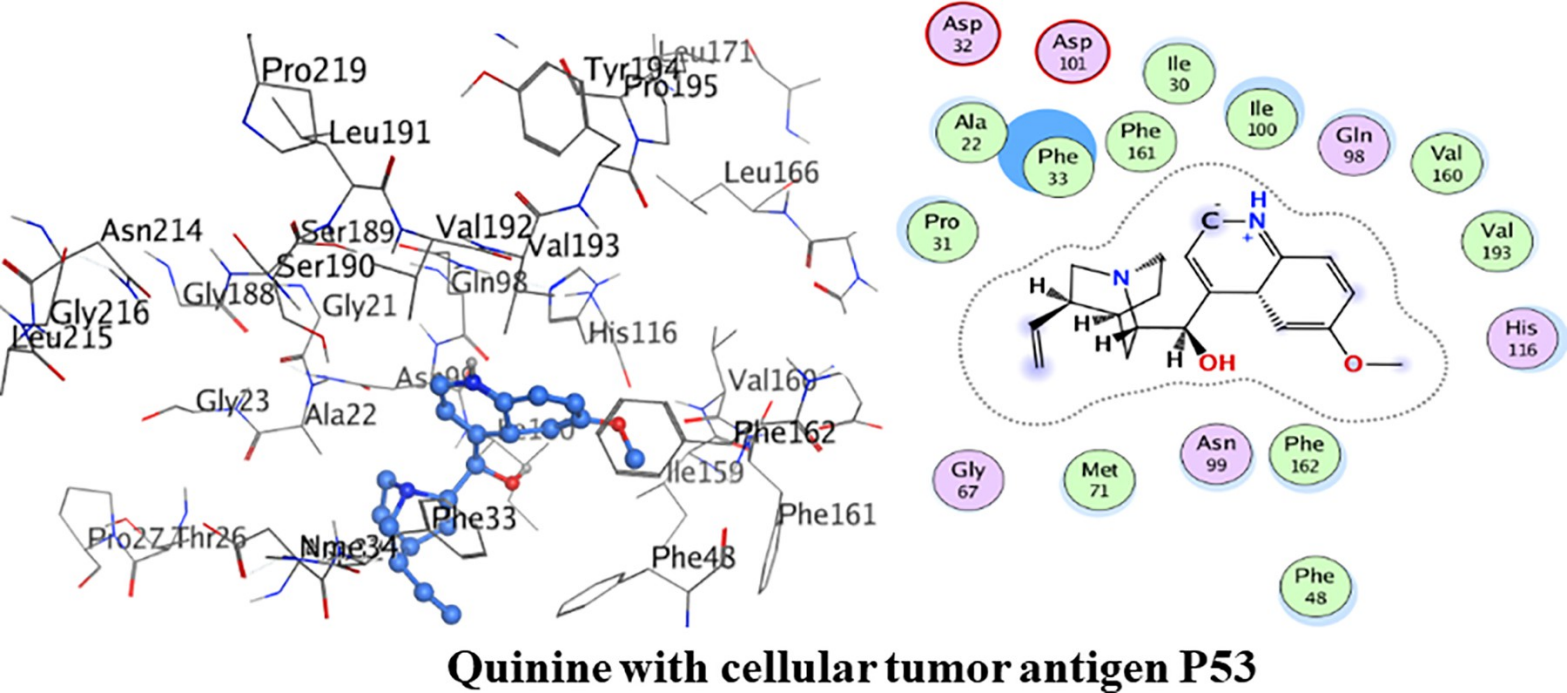
3D and 2D interactions of quinine with active site of cellular tumor antigen P53.

Detailed binding interactions of quinine with cellular tumor antigen P53 are shown in Fig 13 below. Quinine had shown the binding energy of -21.80 kJ/mol. Pro31, Ala22, Phe33, Phe161, Ile30, Ile100, Val160, Val193, Met71 and Phe162 were involved in van der Waals interaction. Amino acid residues Asp32 and Asp101 were the acidic groups encircled with red colour.

#### Molecular docking with caspase-3

**Fig 14:**
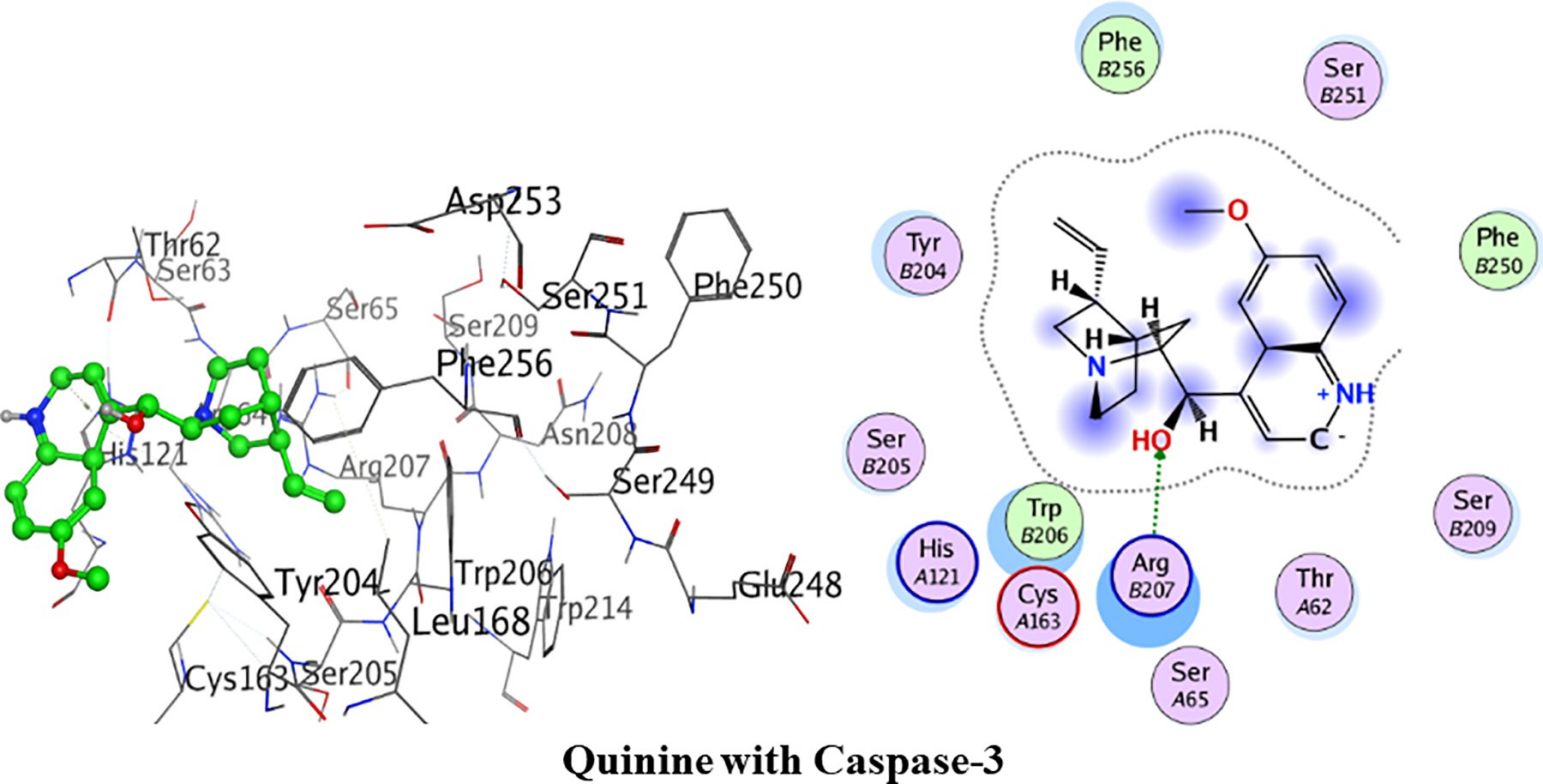
3D and 2D interactions of quinine with active site of caspase-3.

Fig 14 depicts the detailed 3D and 2D interactions. The amino acid residues of the active pocket of caspase 3 are; Trp206, Ser205, Phe256, His121, Tyr204 and Cys163. Quinine had shown the minimum binding energy of -18.96 kJ/mol and showed one hydrogen bond interaction. Amino acid residue Arg207 was observed to form one hydrogen bond interaction with hydroxyl group of quinine. Phe256 and Phe250 were involved in van der Waals interactions.

**Fig 15:**
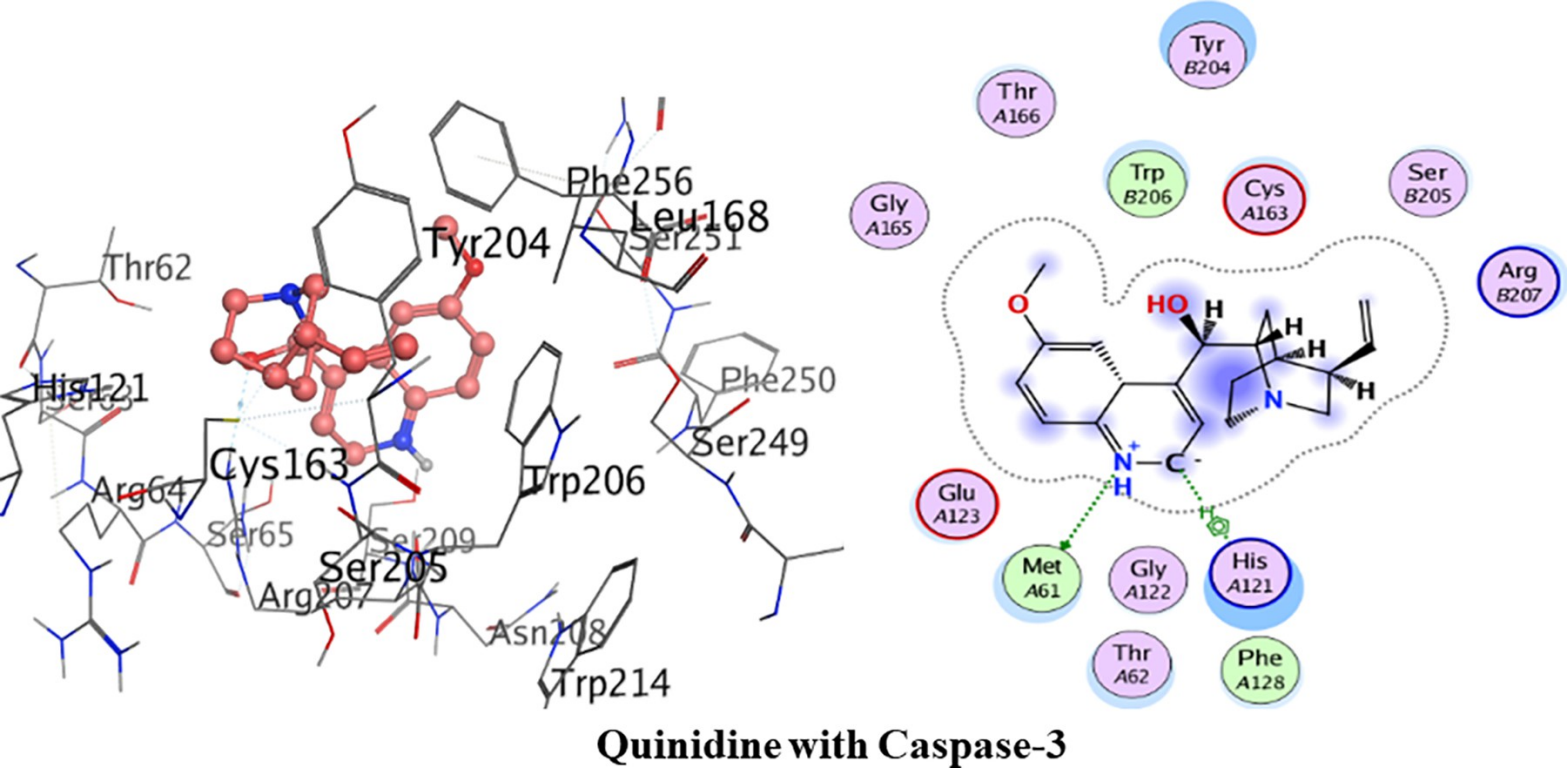
3D and 2D interactions of quinidine with active site of caspase-3.

Detailed binding interactions of quinidine with caspase-3 are shown in Fig 15 below. Quinidine had shown the binding energy of -20.28 kJ/mol. Amino acid residue Met61 was observed in formation of hydrogen bond interaction with amine group of quinidine. One Pi-aryl interaction was formed by amino acid residue His121 with quinidine. Trp206 and Phe128 were involved in van der Waals interaction.

### MD simulations

To calculate the average change in displacement of atoms in regard to a reference frame, Root Mean Square Deviation (RMSD) was used. The value was computed for each and every trajectories frame individually. MD trajectory analysis was also used to determine the root mean square fluctuation (RMSF), and protein–ligand interactions, among other parameters. RMSD for proteins was found as follows: The graphs depicted that how the relative mean squared deviation (RMSD) of a protein was varied over time (left Y-axis). Following the alignment of all protein frames on the reference frame backbone, the RMSD was computed based on atom selection and is shown on the screen.

During the simulation, it is possible to determine the protein’s structure by measuring its relative mean square deviation (RMSD). It is possible to determine if the final variations of the simulation are centered around some type of thermal average structure by doing an RMSD analysis on the data. When it comes to proteins that are both tiny and globular in structure, changes on the order of 1–3 are perfectly suitable. Larger differences, on the other hand, indicate that the protein is experiencing a significant structural change during the simulation. MD simulations that are run for a prolonged period of time reveal considerable structural deviation in the targeted system. Furthermore, it is necessary for attaining equilibrium in RMSD patterns of simulated trajectories. Ligand RMSD is represented on the right Y-axis. The RMSD ligand (right Y-axis) showed the ligand’s stability with respect to the protein and its binding pocket.

**Fig 16:**
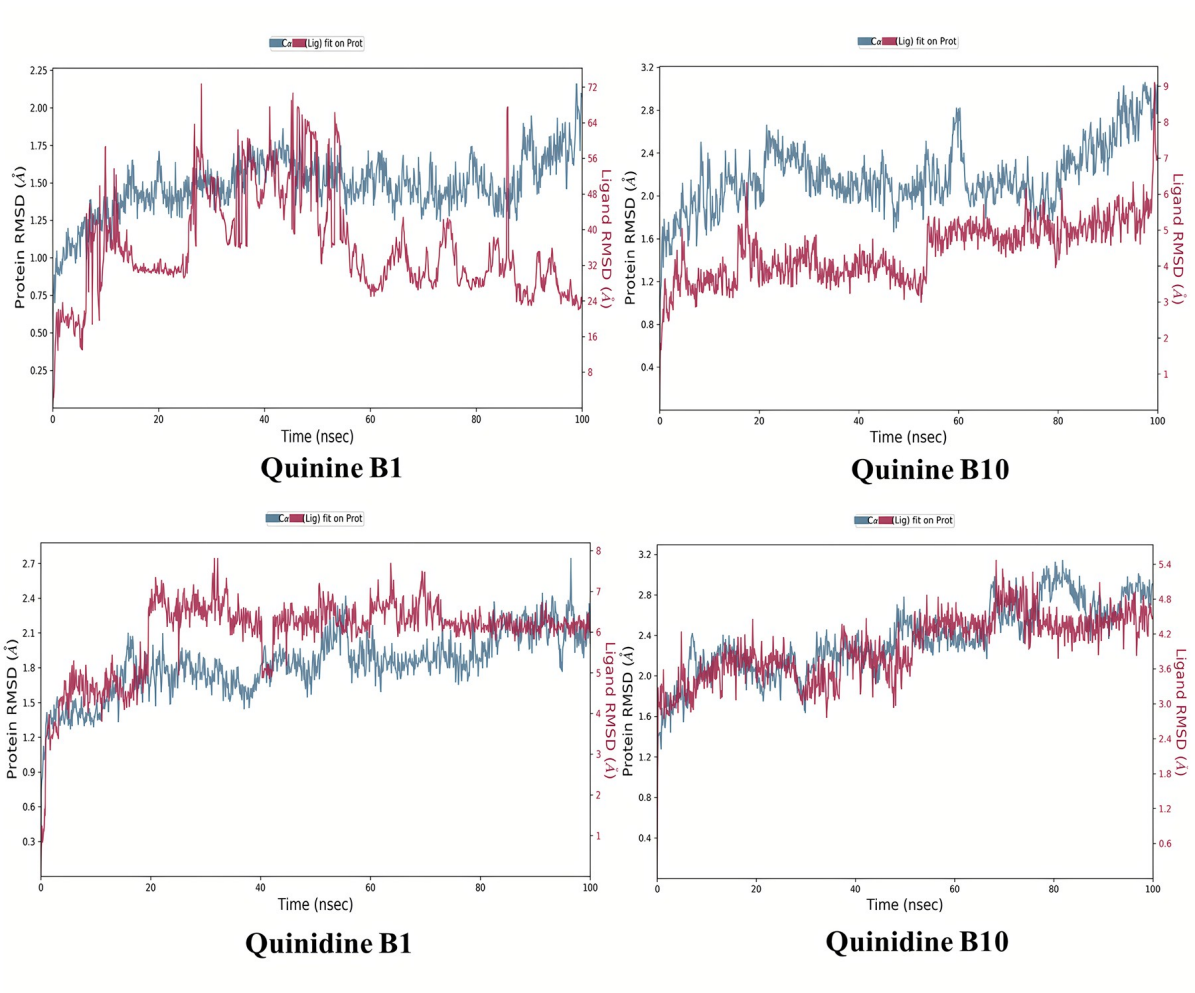
Root mean square deviation (RMSD) of the C-alpha atoms of AKR1B1-Quinine, AKR1B1-Quinidine; AKR1B10-Quinine, AKR1B10-Quinidine complexes with time. The left Y-axis shows the variation of protein RMSD through time. The right Y-axis shows the variation of ligand RMSD through time.

The RMSD of ligand heavy atoms was computed and displayed in this manner after a protein-ligand combination is line up on the reference protein backbone, as shown in the Fig 16. In situations where reported values are much greater than the protein’s standard deviation, it is possible to assume that the ligand has dispersed away from its initial binding site, which is consistent with previous findings.

**Fig 17:**
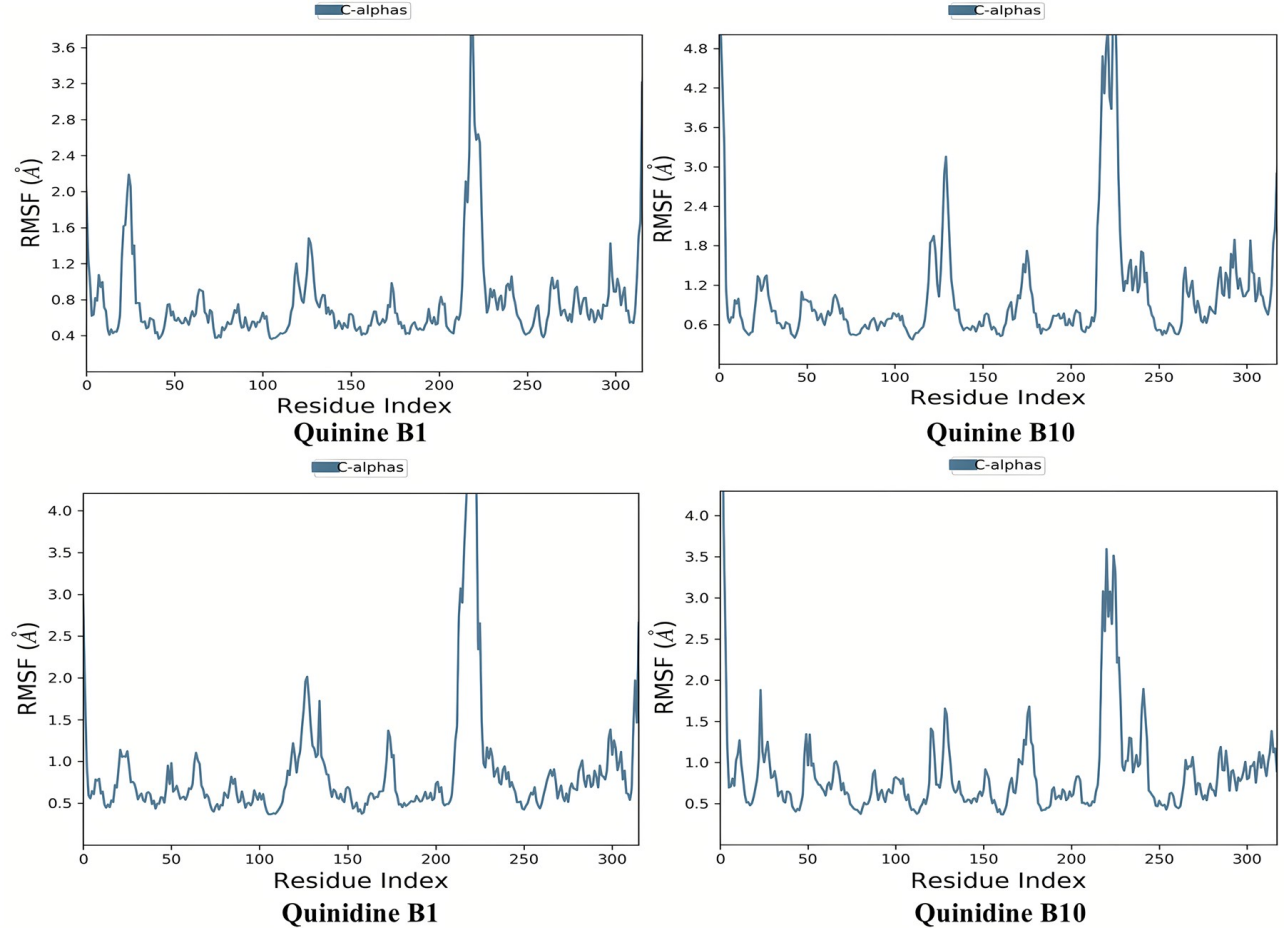
Residue wise Root Mean Square Fluctuation (RMSF) of the C-alpha atoms of AKR1B1-Quinine, Quinidine; AKR1B10-Quinine, Quinidine complexes with time.

Fig 16 illustrates the evolution of the RMSD for the apoprotein C-alpha atoms over time. The RMSD plot of the AKR1B1-Quinine, AKR1B1-Quinidine; AKR1B10-Quinine, AKR1B10-Quinidine indicates variation but get stability. The RMSD fluctuations for the goal remain within 2.0 throughout simulation, which is acceptable but in case of quinidine it fluctuates above 2.0. Between 50 and 60 ns, its root mean square deviation was slightly larger. It stabilized around 60 ns. The simulation results indicate that the ligands were tightly bound to the receptor’s binding site. Root Mean Square Fluctuation (RMSF) of the C-alpha atoms of AKR1B1-Quinine, Quinidine; AKR1B10-Quinine, Quinidine complexes with time are shown in Fig 17. Low RMSF values of the binding site residues demonstrate the stability of ligand binding to the protein. The RMSF of both the ligands revealed some oscillations which demonstrated their dynamical shift at their binding domain in respective proteins. The atoms of AKR1B10-Quinine and AKR1B1-Quinidine displayed more oscillations.

**Fig 18:**
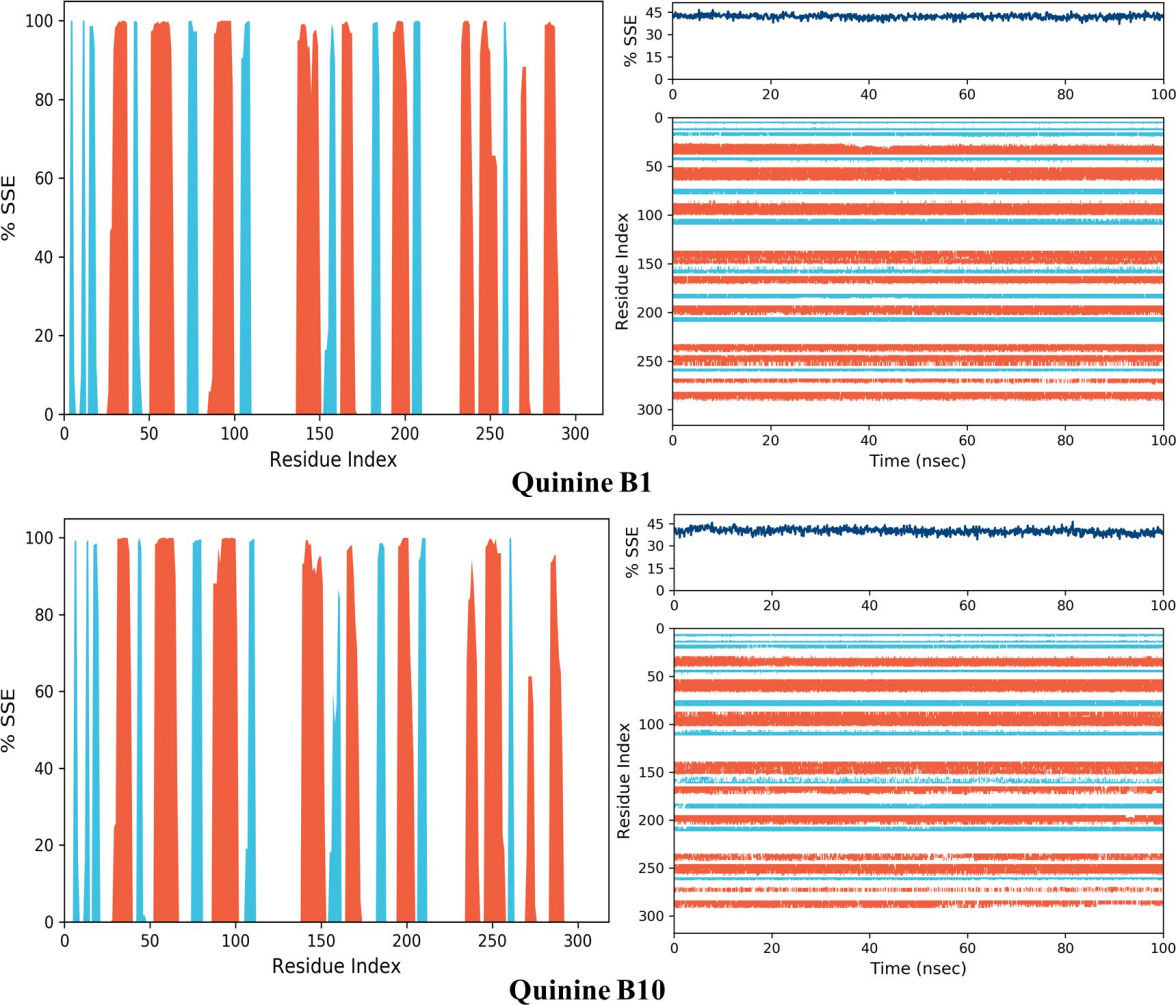
AKR1B1, AKR1B10 bound with quinidine secondary structure element distribution by residue index. The colours red and blue represent alpha helices and beta strands, respectively.

**Fig 19:**
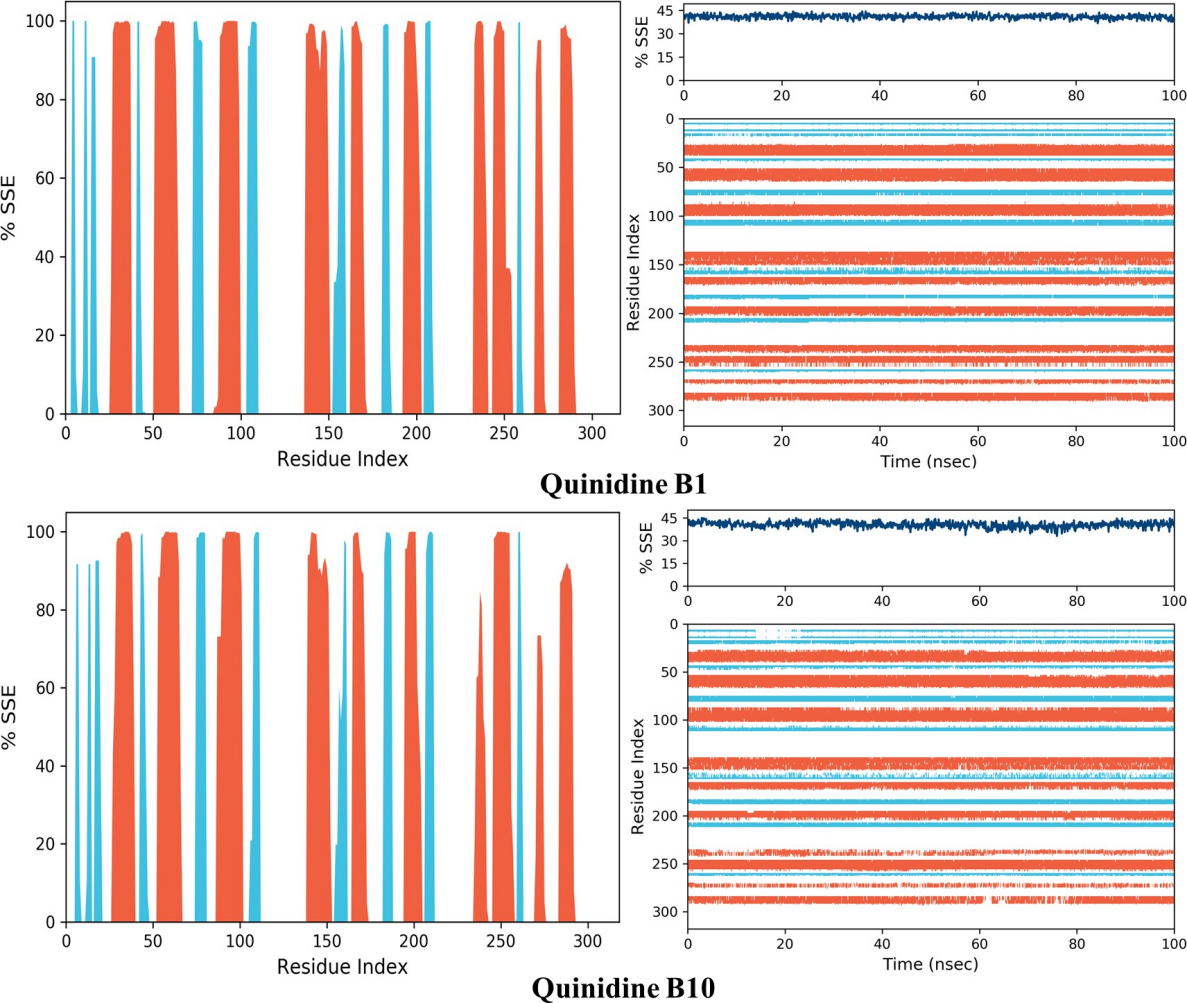
AKR1B1, AKR1B10 bound with quinine secondary structure element distribution by residue index. The colours red and blue represent alpha helices and beta strands, respectively.

The alpha helices and beta strands, which are secondary structural elements, are monitored throughout the simulations (SSE). The distribution of SSE by residue index throughout the protein structure is shown in the above graph. The bottom plot displays the SSE assignment for each residue over time and is presented in Figs 18 and 19. The top image shows the SSE composition for each trajectory frame during simulation.

### ADMET properties

LogP is used to determine a compound’s hydrophilicity; if the LogP value is negative, the compound is hydrophilic. In this research, compounds are lipophilic as they have positive value of LogP. Reduced hydrophilicity (higher LogP values) results in insufficient solubility and absorption. The LogS number indicates solubility: the lower the LogS value, the greater the solubility, which increases absorption. For drugs with CNS activity, the optimal lipophilicity for blood–brain barrier penetration is a LogD≤2. A LogD greater than 4 is considered inappropriate for a central nervous system (CNS) medication. Calculation of the topological polar surface area (TPSA) for the purpose of forecasting the oral absorption of drug-like compounds. A greater TPSA value indicates decreased membrane permeability. Thus, a lower TPSA level was acceptable for drug-likeness. The TPSA value should be low for optimal CNS diffusion. A derivative is deemed to be adequately bioavailable if it has a TPSA of 70. The findings indicated better TPSA values. The number of hydrogen bond donors (nHD) is equal to the total of all OHs and NHs, whereas the number of hydrogen bond acceptors (nHA) is equal to the sum of all nitrogen and oxygen atoms with no positive charge. nHA 0–12 and nHD 0–7 are the optimal ranges (Table 4).

**Table 4:** Physicochemical properties of quinolone derivatives.

PHYSICOCHEMICAL PROPERTIES								
	Molecular Weight	Density	nHA	nHD	TPSA	LogS	LogP	LogD
Quinidine	324.18	0.942	4	1	45.59	-2.454	2.996	2.796
Quinine	324.18	0.942	4	1	45.59	-2.852	2.871	2.767

In terms of absorption and distribution, a high HIA value indicates that the substance will be absorbed more readily from the gastrointestinal system. According to Table 5, derivatives rapidly penetrate the intestinal membrane, increasing the blood plasma concentration. The calculated BBB value indicated that the majority of substances easily pass across the BBB barrier due to their natural lipophilicity. 90% plasma protein binding should be achieved with drugs. It has a poor therapeutic index at higher concentrations. Our compounds demonstrated increased binding to plasma proteins. Almost all substances were found to be both substrate and inhibitor when the efflux via P-glycoprotein (P-gp) was calculated. The volume of distribution (Vd) of a medicine refers to the ratio of its concentration in plasma to its total amount in the body. 0.04-20L/kg is the optimal range. All derivatives chosen are within this ideal range. In vitro human intestinal permeability is calculated using the Caco-2 cell monolayer model as a surrogate. Caco-2 permeability is optimal when it is more than -5.15 log units.

**Table 5:** ADMET properties of the quinolone derivatives.

ABSORPTION & DISTRIBUTION PROPERTIES								
	VOLUME OF DISTRIBUTION (VD)	HUMAN INTESTINAL ABSORPTION (HIA)	CACO-2 PERMEABILITY	BLOOD BRAIN BARRIER (BBB) & BLOOD-PLACENTA BARRIER (BPB)	PLASMA PROTEIN BINDING (PPB)	PGP-INHIBITOR	P-GLYCOPROTEIN SUBSTRATE (PGP-SUBSTRATE)	MDCK PERMEABILITY
Quinidine	2.595	0.00	-4.738	0.573	76.28%	0.998	0.9	1.5e-05
Quinine	2.253	0.01	-4.727	0.923	80.73%	0.999	0.98	1.8e-05
**METABOLISM**							**EXCRETION**	
	**CYP1A2 inhibitor**	**CYP2C19 Inhibitor**	**CYP2C9 inhibitor**	**CYP2D6 inhibitor**	**CYP3A4 inhibitor**		**CL**	**T1/2**
Quinidine	0.127	0.082	0.021	0.97	0.19		2.74	0.299
Quinine	0.244	0.088	0.028	0.973	0.32		2.569	0.24
**MEDICINAL PROPERTIES**			**TOXICITY**					
	**Synthetic Accessibility Score**	**Lipinski Rule**	**AMES Toxicity**	**Carcinogenicity**	**Eye Corrosion**	**Eye Irritation**	**Respiratory Toxicity**	
Quinidine	4.406	Accepted	0.226	0.322	0.003	0.059	0.95	
Quinine	4.406	Accepted	0.318	0.616	0.003	0.067	0.954	
**TOX21 PATHWAY**								
	**NR-AR**		**NR-AR-LBD**		**NR-ER**		**Antioxidant Response Element**	
Quinidine	0.399		0.005		0.462		0.137	
Quinine	0.781		0.02		0.633		0.183	

MDCK cells are employed to investigate drug efflux and active transport, most commonly via P-glycoprotein efflux. All of our derivatives have a low to moderate MDCK permeability. <2 has a low permeability, 2–20 has a medium permeability, and >20 has a high permeability. There are three levels of drug clearance rates: high (>15), moderate (5–15), and low (less than 5). When it comes to therapeutic characteristics and toxicity, the AMES toxicity test is used to determine whether or not a chemical is mutagenic. None of the derivatives are carcinogenic. The ease with which drug-like molecules can be produced is measured using the synthetic accessibility score (SAscore). SAscore 6 is straightforward to synthesize. All of our derivatives were rather straightforward to produce. Additionally, the toxicity profile of the compounds was investigated. According to toxicity risk assessment, the proposed chemical has a lower toxicity profile. According to the projected results, the compounds were not corrosive or irritating to the eyes. They had a lower carcinogenic and respiratory toxicity profile. Analyzing NR-AR predicts whether a derivative stimulates or inactivates the androgen receptor. Each of our compounds acted as an activator. By analyzing NR-AR-LBD, we may anticipate whether or not the androgen receptor ligand-binding domain is activated. Each of our compounds acted as an activator. Analyzing NR-ER provides insight into whether the estrogen receptor is activated or inactivated. All of our compounds were designed to behave as activators. The antioxidant response element is referred to as SR-ARE. All of our derivatives were SR-ARE activators.

## Conclusion

This is the first work to describe quinolone alkaloids as drug candidates for the AKR1B1 and AKR1B10 receptors, which was done using a blend of virtual screening and rationalized insights into the reactivity and stability of complexes gained using rigorous *in silico* techniques. DFT results showed that they perform substantially better and have reactive characteristic. The docking data from both softwares indicated that our compounds had higher binding energies than the co-crystal ligand (NAP). Additionally, ADMET characteristics indicated that the chosen compounds possessed drug-like effects.

Quinine was shown to be an effective inhibitor of AKR1B10, whilst Quinidine was found to be a more strong inhibitor of AKR1B1. The stability and dynamics of the complexes (Quinine and Quinidine) with AKR1B1 and AKR1B10 in the aqueous medium were ultimately confirmed by the outcomes of MD simulations. With the help of these powerful aldo-keto reductase inhibitors, it may be possible to synthesize potential new pharmaceuticals that cure colon cancers associated with aberrant expression of the AKR1B1 or AKR1B10 protein.

## Supporting information

S1 Fig Protein-ligand (AKR1B1-Quinine, Quinidine; AKR1B10-Quinine, Quinidine) contact histogram (H-bonds, hydrophobic, ionic, water bridges). (TIF)

S2 Fig Ligand atom interactions with the protein residues. (AKR1B1-Quinine, Quinidine; AKR1B10-Quinine, Quinidine).
(TIF)

S3 Fig Ligand properties. (Quinine, Quinidine ligand).
(TIF)

S4 Fig Ligand torsion profile (Quinidine). (TIF)

S5 Fig Ligand torsion profile (Quinine). (TIF)
