# Supplementary figures and images for: In-silico Investigations of quinine and quinidine as potential Inhibitors of AKR1B1 and AKR1B10: Functional and structural characterization
Source: PLoS One. 2022 Oct 27;17(10):e0271602. doi: 10.1371/journal.pone.0271602 (PMC9612481; doi:10.1371/journal.pone.0271602)

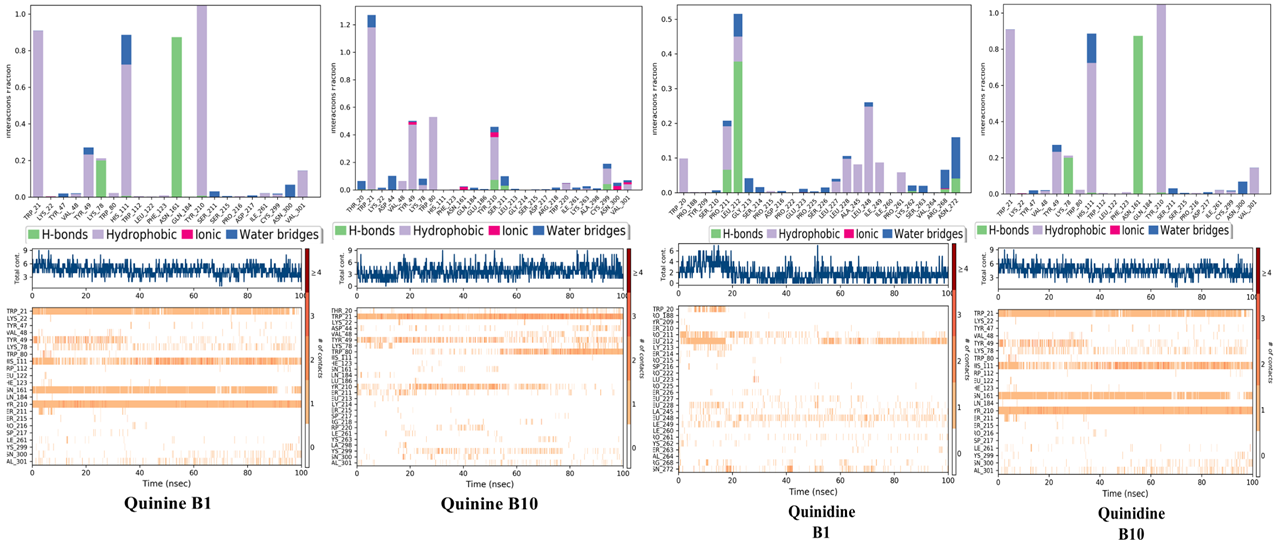

Supplement: S1 Fig — (TIF) [file pone.0271602.s001.tif]

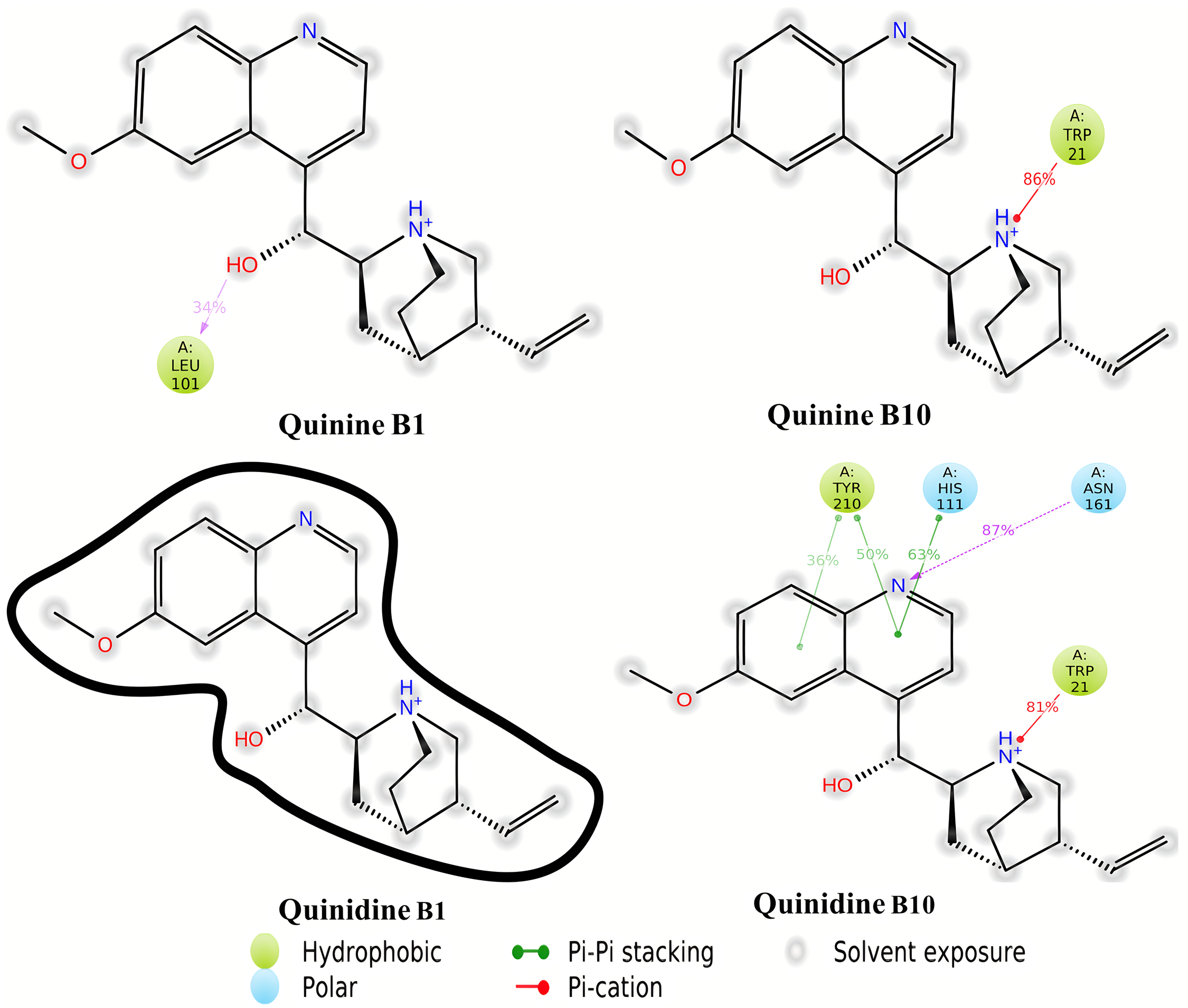

Supplement: S2 Fig — (AKR1B1-Quinine, Quinidine; AKR1B10-Quinine, Quinidine). (TIF) [file pone.0271602.s002.tif]

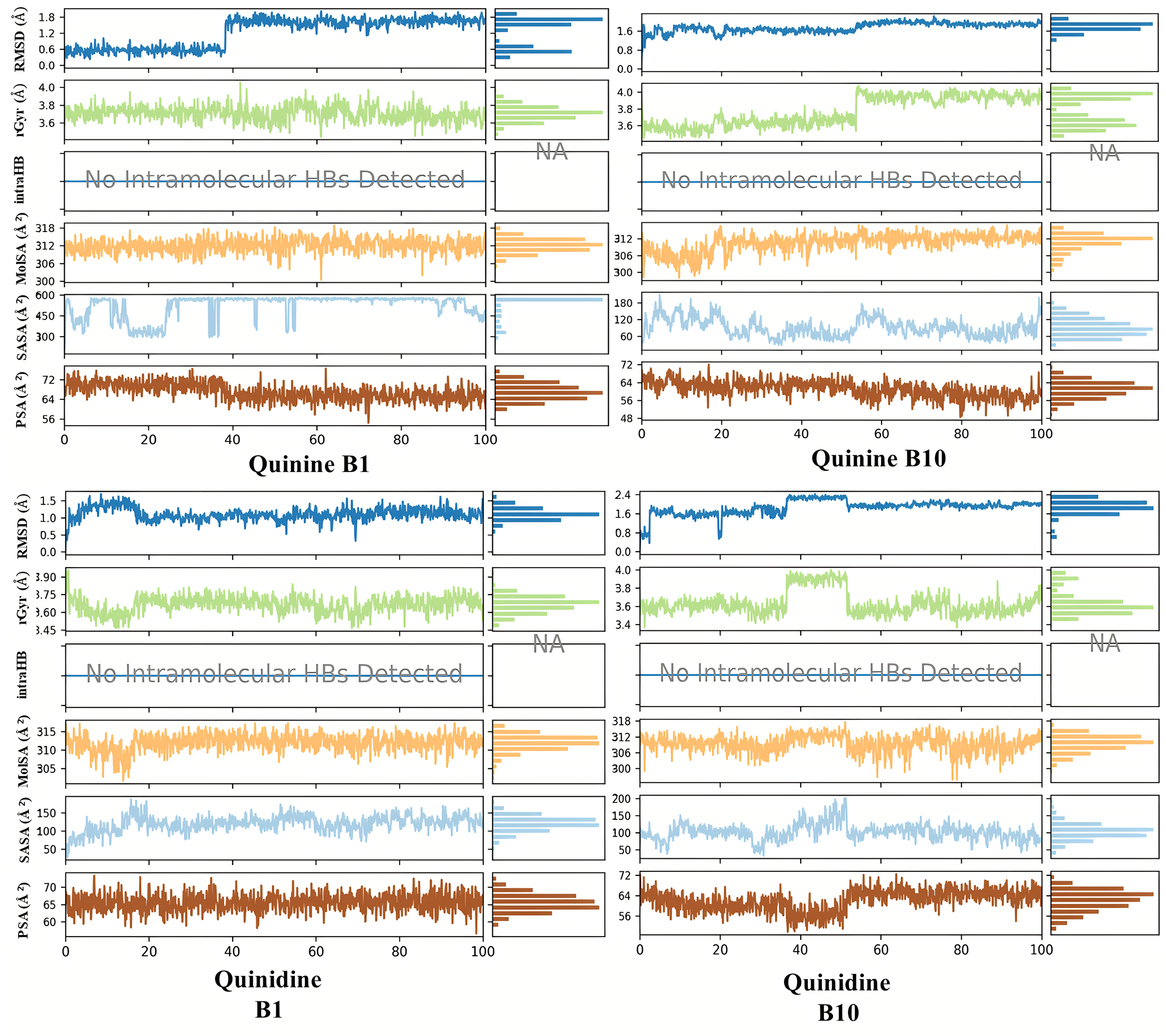

Supplement: S3 Fig — (Quinine, Quinidine ligand). (TIF) [file pone.0271602.s003.tif]

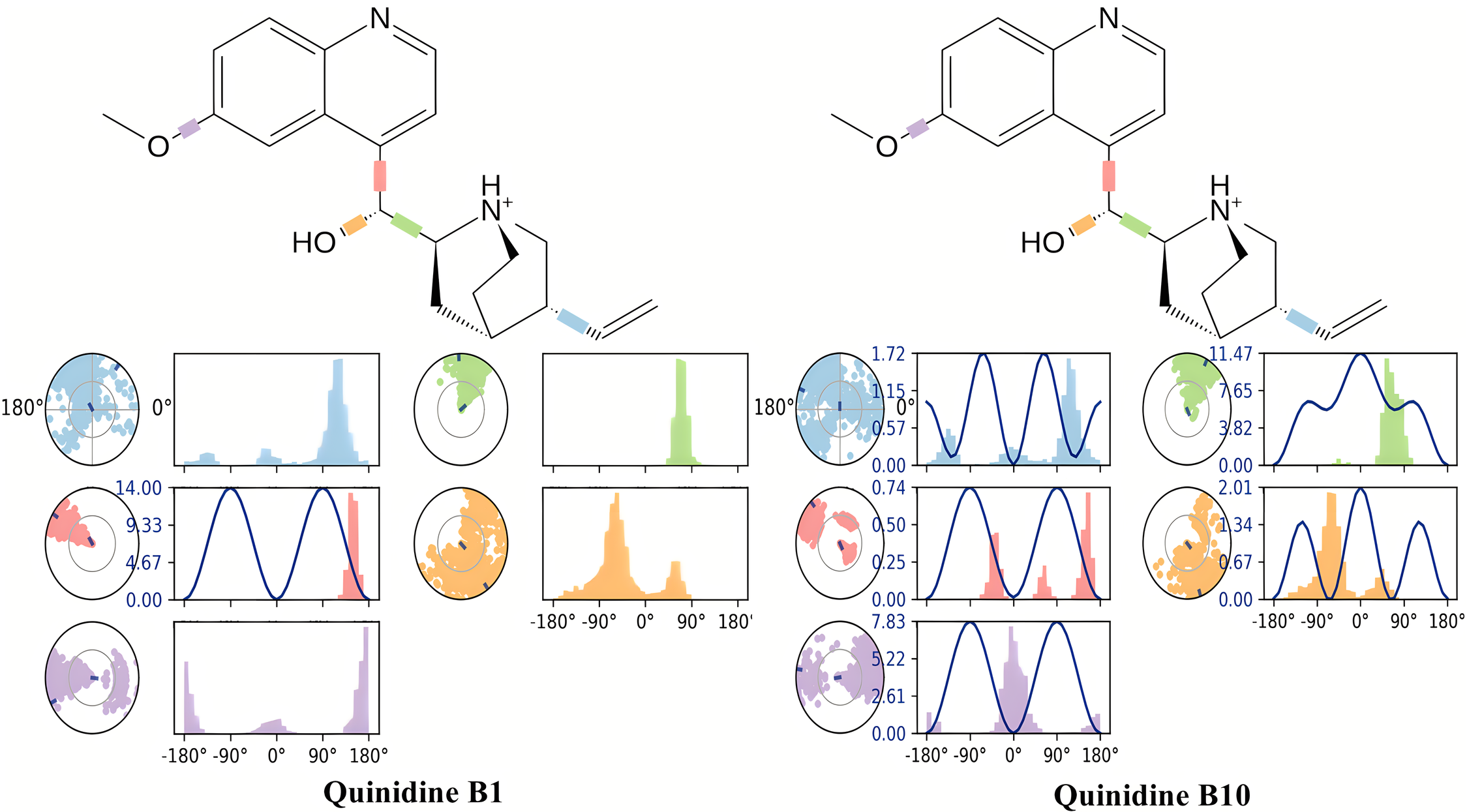

Supplement: S4 Fig — (TIF) [file pone.0271602.s004.tif]

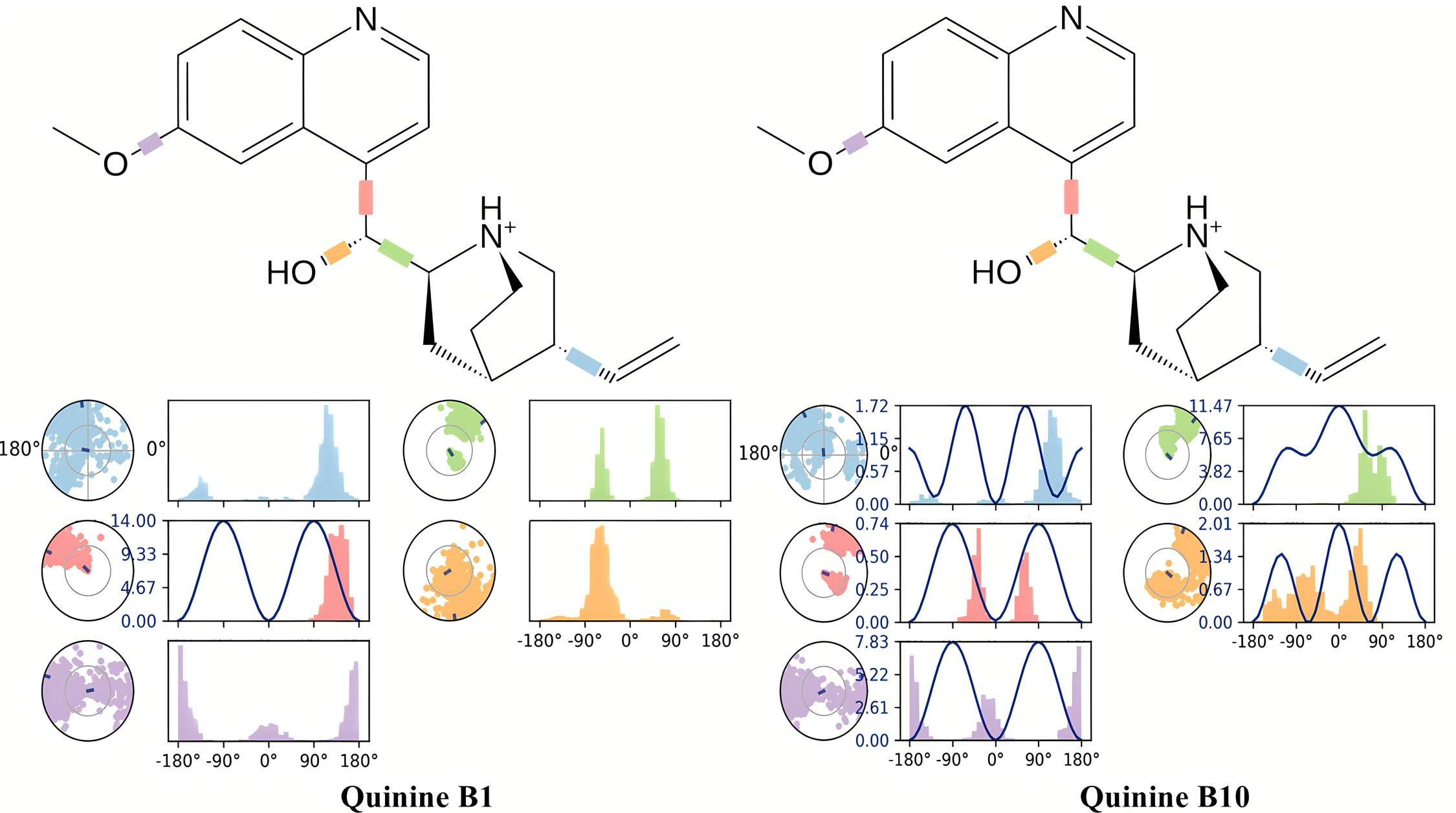

Supplement: S5 Fig — (TIF) [file pone.0271602.s005.tif]
