# Supplementary material for: In-silico Investigations of quinine and quinidine as potential Inhibitors of AKR1B1 and AKR1B10: Functional and structural characterization
Source: PLoS One. 2022 Oct 27;17(10):e0271602. doi: 10.1371/journal.pone.0271602 (PMC9612481; doi:10.1371/journal.pone.0271602)
Supplement: Attachment — Submitted filename: Reviewer report.doc [file pone.0271602.s006.doc]

**In-silico Investigations of Quinine and Quinidine as potential Inhibitors of AKR1B1 and AKR1B10: Functional and Structural Characterization**

**Reviewer #1: Referee Report**

• Abstract section is very weak and this section should be more specific.

**Response:** The abstract has been revised, as suggested

• Why B3LYP/SVP can you explain?

**Response:** B3lyp is a functional, which includes exact exchange and GGA corrections in addition to LDA electron-electron and electron-nuclei energy. The weights of the parts were fit to reproduce geometry of a test suite of small molecules. As such use of b3lyp for calculations with heavier atoms is questionable. B3LYP is generally faster than most Post Hartree-Fock techniques and usually yields comparable results. It is also fairly robust for a DFT method. On a more fundamental level, it is not as heavily parameterized as other hybrid functionals, having only 3 where as some have up to 26. Becke's original paper is one of the most cited papers (I think it's number 8) of all time, so B3LYP is well established in the literature and people are less likely to complain about your choice versus a newer functional.

• Introduction section, FMO, MEP, ADMET, drug-likeness of the title molecule should be expanded with new references such as

1- Synthesis, spectroscopic characterization, DFT, molecular docking and in vitro antibacterial potential of novel quinoline derivatives, Journal of Molecular Structure, Volume 1246, 15 December 2021, 131217
2- Quantum computational, Spectroscopic Investigations on N-(2-((2-chloro-4,5-dicyanophenyl)amino)ethyl)-4-methylbenzenesulfonamide by DFT/TD-DFT with Different Solvents, Molecular Docking and Drug-Likeness Researches, Colloids and Surfaces A Physicochemical and Engineering Aspects 638:128311

3- New Heterocyclic Compound as Carbon Steel Corrosion Inhibitor in 1 M H2SO4, High Efficiency at Low Concentration: Experimental and Theoretical Studies <https://doi.org/10.1080/01694243.2022.2034588>

**Response:** These references have been incorporated in the revised manuscript

• The resolutions of the all figures should be increased.

**Response:** The resolution of all figures has been increased, as suggested

• I can not see the label of Fig. 1 should be corrected.

**Response:** Figure 1 has been revised

• Figure 2 should be transparent form and the labels of atoms should be added.

**Response:** Figure 2 has been revised

• Figure 3 should be revised.

**Response:** Figure 3 has been revised

• Figure 4-9 should be revised very complex.

**Response:** Figures have been revised

• The resolutions of the figures should be increased.

**Response:** The resolution of all figures has been increased, as suggested

• Why these receptors were select for the molecular docking should be clarified.

**Response:** As our work aims to identify potent aldo-keto reductase inhibitors that would help to treat the colon cancer, in which the higher levels of AKR1B1 is observed [1]. AKR1B10 was selected just to confirm whether the inhibitor is selective for AKR1B1 or not.

Reference: [1] Taskoparan B, Seza EG, Demirkol S, Tuncer S, Stefek M, Gure AO, Banerjee S, Opposing roles of the aldo-keto reductases AKR1B1 and AKR1B10 in colorectal cancer. Cellular Oncology, 2017:40(6):563-578.

• Why both Autodock and MOE are needed for calculations?

**Response:** To check the reliability of both softwares. Thus, AutoDock was proved to be the best among the two softwares because it showed less dispersion as compared to MOE on repeated docking protocols.

• If possible experimental activity should be added.

**Response:** Yes the reviewer is right that computational work required experimental evidence. In our case based on available resources, we have provided the extensive computational work.We have already planned for our upcoming publication and both enzymes are already in purchase demand. All the authors are thankful to the reviewer in advance for considering this study based on computational data. Hope the reviewer will understand our situation.

• Conclusion section should be revised.

**Response:** Conclusion has been revised

MINOR REVISION

Reviewer #2: The paper is written in a style that is not typical of well conceived scientific literature. The main drawback of the paper is that in general there are many values calculated, but no discussion of what they mean and how they compare to other materials. Therefore the reader must find a meaning by him/herself of the computed quantities. On the other hand, many times there are explanations about the physical and chemical meanings of some quantities, which are well known in the literature and should not be explained again. A deep revision of the style of the paper is needed.

Some required minor changes are reported in the following:

- Revise the first sentence of the introduction

**Response:** This sentence has been revised

- Molecular Dynamics simulations: "For 100 nanoseconds, MD simulations were carried out for both the complexes by using the same protocol as reported earlier". Add a reference

**Response:** Thank You for suggesting, the reference was already added but not cited properly. Now it has been corrected

- remove the word "showing" from the legends of the figures

**Response:** Corrected as suggested

- revise the first sentence after the caption of Figure 1

**Response:** This sentence has been revised

- In the sentence just before Table 1, which symmetry constraints are used?

**Response:** It was a typographical mistake, no symmetry constraints were applied

- Table 1 seems to indicate that calculations in the solvent phase are performed in water, but the fourth sentence after Table 1 seems to indicate they are performed in methanol.

**Response:** It was a typographical mistake which is corrected in revised manuscript. Water was used as a solvent

- Frontier molecular orbitals: "In gas phase, both the compounds Quinidine and Quinine had shown the same energy gap of 0.15 eV. On contrast in solvent phase (water), again both the compounds had shown the same energy gap of 0.15 eV." There is no contrast.

**Response:** It was a typographical mistake and has been corrected in revised version

- Third line before Molecular electrostatic potential: was -> were

**Response:** Corrected as suggested

- MD simulations Line 9: "the protein's structural structure" revise

**Response:** Corrected

- rephrase the part "It is critical for your simulation to achieve a point of convergence, as well as for your residual standard deviation (RMSD) to settle at a constant level. It's likely that your system hasn't achieved equilibrium yet, and that your simulation hasn't run for long enough to offer a thorough assessment of the protein's relative molecular weight distribution"

**Response:** Suggested part has been rephrased in the revised manuscript

- "Between 50 and 60 Angstroms, its root mean square deviation was slightly larger". Are the authors sure about the units?"

**Response:** You pointed out right, it is a typo mistake because MD trajectory showed slightly higher RMSD pattern during 50 to 60 nanoseconds of simulation time but mistakenly it was written as 50-60 angstrom.

- Revise the first sentence of conclusions

**Response:** This has been revised

- Rephrase the sentences "To be truthful, it is not viable to repeat computations for the same ligands because the software is quite lengthy. That is why we calculated the RMSD value using the best poses. Additionally, to validate and verify our findings, co-crystal ligand (NAP) was docked alongside ligands."

**Response:** It has been rephrased in revised manuscript

- The conclusions are not conclusions in the sense that they do not show the main results of the study.

**Response:** The conclusion has been revised, as suggested

- Quinidine and quinine in Figure 1 look identical. Please, point out the difference.

**Response:** You are right, they look alike in optimized structures because they are isomers.

- Figure S3: labels and numbers superimpose

**Response:** Figure S3 has been revised

- Figure S4: label, numbers and plots superimpose

**Response:** Figure S4 has been revised

Reviewer #3:

This article contains information and investigation of Quinine and Quinidine as potential Inhibitors of AKR1B1 and AKR1B10. It is not determined inconsistency for dual publication, research ethics, and publication ethics. I think that this manuscript is appropriate to publish in the PLOS ONE.

Reviewer #4: Dear Authors,

I have reviewed your manuscript, and I am expressing my positive feedback. Your study is interesting for the readers of the PLOS One journal, and the obtained results are promising. However, there is space for improvements, so I am requesting revisions according to the following comments:

• You should elaborate why did you use SVP basis set, when the def2-TZVP is mostly recommended? Your molecular are not large and the def2-TZVP basis set should have been used.

**Response:** You are correct, def2-TZVP is the most valuable functional but we donot have enough computational resources to test this. For saving the time and computational cost we recommended small SVP basis set.

• Why the acronym of density functional theory is “DFTs”?

**Response:** This is a mistake; the correct acronym is DFT and replaced/corrected in the updated paper.

• In the chapter dealing with the global reactivity properties, you have only provided numerical values of the quantities, without proper scientific discussion. Also, that chapter doesn’t contain equations how these descriptors have been calculated.

**Response:** Thank you for highlighting this part. We have added the details for these parameters in the revised manuscript.

• Please elaborate why you have performed calculations in both gas and solvent phases?

**Response:** The minima corresponding to the gas-phase and solution (PCM model) are definitely not the same. If you're a purist, you should redo all the calculations using the PCM model to get new minima (checked by frequency calculations) and treat the gas-phase calculations just as a good starting point for PCM ones. Although structural differences are typically very small, they will have an effect on energies. The investigated compounds are biological compounds that mostly work according to the body's environment. Usually energy of stabilization/equilibrium change by using the gas phase and solution phase. So, we used both the gas phase and solvent environment so that we know about these parameters.

• Regarding your calculations, also please insert the information about the convergence criteria for SCF and optimization procedures. If you used the default settings, it is enough to mention that the default settings have been used, but in that case please mention which revision of the Gaussian program was used for calculations.

**Response:** The Gaussian 09 package (Rev.E.01) with default settings was used for all calculations.
