# Supplementary material for: In-silico Investigations of quinine and quinidine as potential Inhibitors of AKR1B1 and AKR1B10: Functional and structural characterization
Source: PLoS One. 2022 Oct 27;17(10):e0271602. doi: 10.1371/journal.pone.0271602 (PMC9612481; doi:10.1371/journal.pone.0271602)
Supplement: Attachment — Submitted filename: PONE-Reviewer Comments-7th June 2022.docx [file pone.0271602.s007.docx]

***Reviewer Decision:***

Major Revision Required

***Reviewer Comments:***

**1.** The abstract has been revised well. The very first line does not convey the message. Rephrase.

**2.** In results and discussion section, the author has given the following paragraph: In gas phase, both the compounds Quinidine and Quinine had shown the same energy gap of 0.15 eV…………. . But have not fully justified the meaning of values or their impact on the reactivity of compounds as inhibitors for the targeted protein. Moreover, I do not find enough explanation in terms of DFT study which indeed is quite powerful tool for giving reliable predictions about structure reactivity pattern besides many other chemical features. In a nutshell, this section seems more like a general theory for DFT rather than case study details and explanations. There is a lot room for improvement in this study.

**3.** The manuscript needs revision in terms of English language and grammar like e.g. In results and discussion section: “Here, is the case with Quinidine and Quinine shows the same significant values as they are the optical isomers of each other.”

&

In introduction section: “From this study, may the quinolone derivatives be proved lead compounds for the treatment of colon cancer by inhibiting aldo-keto reductase (17).”

**4.** There is a lack in connection between the generalization for Global chemical reactivity descriptors values and the values obtained for the test molecules with ultimate effect on reactivity with the targeted proteins. Moreover, authors would have considered predicting the SAR for the inhibitor molecules based on different parameters studied by DFT. A lot much can be improved in DFT section. It seems authors are unable to fully explain/justify the results systematically besides having calculation of values for various useful parameters.

**5.** Authors state in molecular docking section as:” The hydrogen bonding is significant in protein ligand interaction to check the inhibitory action.” That is true but not a universal rule indeed for docked ligands. To rationalize this statement authors, need to justify in their case by giving suitable explanation in terms of structure activity relationship.

**6.** Incomplete explanation of the following statement: “LogP is used to determine a compound's hydrophilicity; if the LogP value is negative, the compound is hydrophilic. Compounds are lipophilic.” Which value type indicates lipophilic behavior, not explained.
