# Supplementary material for: In-silico Investigations of quinine and quinidine as potential Inhibitors of AKR1B1 and AKR1B10: Functional and structural characterization
Source: PLoS One. 2022 Oct 27;17(10):e0271602. doi: 10.1371/journal.pone.0271602 (PMC9612481; doi:10.1371/journal.pone.0271602)
Supplement: Attachment — Submitted filename: PONE-Reviewer Comments-7th June 2022.docx [file pone.0271602.s008.docx]

***Reviewer Decision:***

Major Revision Required

***Reviewer Comments:***

**Reviewer #5:** The manuscript is concise, informative, and well written. The authors have thoroughly shed light on the Quinine and Quinidine as potential inhibitors of AKR1B1 and AKR1B10. All the suggestions of the reviewers are appropriately incorporated. Findings of the study favour Quinidine as a better inhibitor of AKR1B1 and Quinine as a plausible inhibitor of AKR1B10.The article is helpful in the concerned field of pharmacopoeia and rational drug design. Therefore, it is recommendable for publication in the journal. However, authors must check once again for typos and grammar mistakes throughout the manuscript.

**Response:** First of all Thanks to the reviewer for considering the research article. Now all the typos and grammar mistakes have been corrected in the revised manuscript

**Reviewer #6:** The reviewer comments are attached. Author needs to work on the pointed areas by giving suitable justifications and explanations in the manuscript.

**1.** The abstract has been revised well. The very first line does not convey the message. Rephrase.

**Response:** The abstract has been revised as suggested

**2.** In results and discussion section, the author has given the following paragraph: In gas phase, both the compounds Quinidine and Quinine had shown the same energy gap of 0.15 eV…………. . But have not fully justified the meaning of values or their impact on the reactivity of compounds as inhibitors for the targeted protein. Moreover, I do not find enough explanation in terms of DFT study which indeed is quite powerful tool for giving reliable predictions about structure reactivity pattern besides many other chemical features. In a nutshell, this section seems more like a general theory for DFT rather than case study details and explanations. There is a lot room for improvement in this study.

**Response:** The required justifications and corrections have be incorporated in the revised manuscript as suggested.

**3.** The manuscript needs revision in terms of English language and grammar like e.g. In results and discussion section: “Here, is the case with Quinidine and Quinine shows the same significant values as they are the optical isomers of each other.”

&

In introduction section: “From this study, may the quinolone derivatives be proved lead compounds for the treatment of colon cancer by inhibiting aldo-keto reductase (17).”

**Response:** All the grammatical errors have been removed from the revised manuscript

**4.** There is a lack in connection between the generalization for Global chemical reactivity descriptors values and the values obtained for the test molecules with ultimate effect on reactivity with the targeted proteins. Moreover, authors would have considered predicting the SAR for the inhibitor molecules based on different parameters studied by DFT. A lot much can be improved in DFT section. It seems authors are unable to fully explain/justify the results systematically besides having calculation of values for various useful parameters.

**Response:** It has been revised, as suggested.

**5.** Authors state in molecular docking section as:” The hydrogen bonding is significant in protein ligand interaction to check the inhibitory action.” That is true but not a universal rule indeed for docked ligands. To rationalize this statement authors, need to justify in their case by giving suitable explanation in terms of structure activity relationship.

**Response:** The structure activity relationship has been incorporated in the revised manuscript as suggested.

**6.** Incomplete explanation of the following statement: “LogP is used to determine a compound's hydrophilicity; if the LogP value is negative, the compound is hydrophilic. Compounds are lipophilic.” Which value type indicates lipophilic behavior, not explained.

**Response:** It has been explained in the revised manuscript

**Reviewer #7:** Dear editor,

Have a nice day. The authors carried out some In-silico tests for Quinine and Quinidine as potential Inhibitors of AKR1B1 and AKR1B10.

The manuscript lacks novelty in some points

1- That the bases to select Quinine and Quinidine as a potential target for AKR1B1 and AKR1B10? Are these compounds have some sort of similarity with the previously reported AKR1B1 and AKR1B10 inhibitors?? If this is the case, confirm the structural similarity.

**Response:** The detailed rationale has been incorporated in the revised manuscript as suggested

2- The binding modes of Quinine and Quinidine need more clarification. Compare the binding mode of Quinine and Quinidine with that of co-crystallized ligand.

**Response:** The suggestion has been addressed in the revised manuscript

3- In vitro cytotoxicity of Quinine and Quinidine against cancer cell line is necessary.

**Response:** Yes the reviewer is right that computational work required experimental evidence. Our studies are based on available resources. We have put the purchase demand of targeted enzymes and cell lines, and we will consider it in our upcoming publications. Hope the reviewer will understand our situation and accept this appology. However, a detailed insilico studies against cancer markers have been added to support our results.

4- In vitro enzymatic inhibition is necessary.

**Response:** The reviewer gave a very fruitful suggestion and we agree that without in-vitro justification out results are partially validated. We have provided more in-silico justifications that our compounds have strong anticancer potential. We have put purchase demand of our targeted Enzymes and they are in process. This will take time and at this stage, authors of this study are thankful in advance to the reviewer to consider our justifications of this point. We are working on more compounds and we will incorporate in-vitro studies in our upcoming publication along with the justification of current compounds.
