# Supplementary material for: In-silico Investigations of quinine and quinidine as potential Inhibitors of AKR1B1 and AKR1B10: Functional and structural characterization
Source: PLoS One. 2022 Oct 27;17(10):e0271602. doi: 10.1371/journal.pone.0271602 (PMC9612481; doi:10.1371/journal.pone.0271602)
Supplement: Peer review history

# Peer review: Decision Letter 0

**DOI:** 10.1371/journal.pone.0271602.r001

**Authors:** Joazaizulfazli Jamalis

6 May 2022

PONE-D-22-10464In-silico Investigations of Quinine and Quinidine as potential Inhibitors of AKR1B1 and AKR1B10: Functional and Structural CharacterizationPLOS ONE

Dear Dr. Ejaz,

Thank you for submitting your manuscript to PLOS ONE. After careful consideration, we feel that it has merit but does not fully meet PLOS ONE’s publication criteria as it currently stands. Therefore, we invite you to submit a revised version of the manuscript that addresses the points raised during the review process.

Please submit your revised manuscript by Jun 19 2022 11:59PM. If you will need more time than this to complete your revisions, please reply to this message or contact the journal office at plosone@plos.org. When you're ready to submit your revision, log on to https://www.editorialmanager.com/pone/ and select the 'Submissions Needing Revision' folder to locate your manuscript file.

Please include the following items when submitting your revised manuscript:

- A rebuttal letter that responds to each point raised by the academic editor and reviewer(s). You should upload this letter as a separate file labeled 'Response to Reviewers'.
- A marked-up copy of your manuscript that highlights changes made to the original version. You should upload this as a separate file labeled 'Revised Manuscript with Track Changes'.
- An unmarked version of your revised paper without tracked changes. You should upload this as a separate file labeled 'Manuscript'.

If you would like to make changes to your financial disclosure, please include your updated statement in your cover letter. Guidelines for resubmitting your figure files are available below the reviewer comments at the end of this letter.

If applicable, we recommend that you deposit your laboratory protocols in protocols.io to enhance the reproducibility of your results. Protocols.io assigns your protocol its own identifier (DOI) so that it can be cited independently in the future. For instructions see: https://journals.plos.org/plosone/s/submission-guidelines#loc-laboratory-protocols. Additionally, PLOS ONE offers an option for publishing peer-reviewed Lab Protocol articles, which describe protocols hosted on protocols.io. Read more information on sharing protocols at https://plos.org/protocols?utm_medium=editorial-email&utm_source=authorletters&utm_campaign=protocols.

We look forward to receiving your revised manuscript.

Kind regards,

Joazaizulfazli Jamalis

Academic Editor

PLOS ONE

Journal Requirements:

When submitting your revision, we need you to address these additional requirements.

1. Please ensure that your manuscript meets PLOS ONE's style requirements, including those for file naming. The PLOS ONE style templates can be found at 

https://journals.plos.org/plosone/s/file?id=wjVg/PLOSOne_formatting_sample_main_body.pdf and 

https://journals.plos.org/plosone/s/file?id=ba62/PLOSOne_formatting_sample_title_authors_affiliations.pdf.

2. Thank you for stating the following in the Acknowledgments Section of your manuscript: 

[This work was also supported by King Khalid University through a grant (RCAMS/KKU/G001/21) under the Research Center for Advanced Materials Science (RCAMS) at King Khalid University, Saudi Arabia.]

We note that you have provided funding information that is not currently declared in your Funding Statement. However, funding information should not appear in the Acknowledgments section or other areas of your manuscript. We will only publish funding information present in the Funding Statement section of the online submission form. 

Please remove any funding-related text from the manuscript and let us know how you would like to update your Funding Statement. Currently, your Funding Statement reads as follows: 

 [The author(s) received no specific funding for this work.]

Please include your amended statements within your cover letter; we will change the online submission form on your behalf.

3. PLOS requires an ORCID iD for the corresponding author in Editorial Manager on papers submitted after December 6th, 2016. Please ensure that you have an ORCID iD and that it is validated in Editorial Manager. To do this, go to ‘Update my Information’ (in the upper left-hand corner of the main menu), and click on the Fetch/Validate link next to the ORCID field. This will take you to the ORCID site and allow you to create a new iD or authenticate a pre-existing iD in Editorial Manager. Please see the following video for instructions on linking an ORCID iD to your Editorial Manager account: https://www.youtube.com/watch?v=_xcclfuvtxQ.

4. We note you have included a table to which you do not refer in the text of your manuscript. Please ensure that you refer to Table 4 in your text; if accepted, production will need this reference to link the reader to the Table.

[Note: HTML markup is below. Please do not edit.]

Reviewers' comments:

Reviewer's Responses to Questions

**Comments to the Author**

1. Is the manuscript technically sound, and do the data support the conclusions?

The manuscript must describe a technically sound piece of scientific research with data that supports the conclusions. Experiments must have been conducted rigorously, with appropriate controls, replication, and sample sizes. The conclusions must be drawn appropriately based on the data presented. 

Reviewer #1: Yes

Reviewer #2: Partly

Reviewer #3: No

Reviewer #4: Yes

**********

2. Has the statistical analysis been performed appropriately and rigorously? 

Reviewer #1: Yes

Reviewer #2: N/A

Reviewer #3: No

Reviewer #4: N/A

**********

3. Have the authors made all data underlying the findings in their manuscript fully available?

The PLOS Data policy requires authors to make all data underlying the findings described in their manuscript fully available without restriction, with rare exception (please refer to the Data Availability Statement in the manuscript PDF file). The data should be provided as part of the manuscript or its supporting information, or deposited to a public repository. For example, in addition to summary statistics, the data points behind means, medians and variance measures should be available. If there are restrictions on publicly sharing data—e.g. participant privacy or use of data from a third party—those must be specified.

Reviewer #1: Yes

Reviewer #2: Yes

Reviewer #3: Yes

Reviewer #4: Yes

**********

4. Is the manuscript presented in an intelligible fashion and written in standard English?

PLOS ONE does not copyedit accepted manuscripts, so the language in submitted articles must be clear, correct, and unambiguous. Any typographical or grammatical errors should be corrected at revision, so please note any specific errors here.

Reviewer #1: Yes

Reviewer #2: No

Reviewer #3: Yes

Reviewer #4: Yes

**********

5. Review Comments to the Author

Please use the space provided to explain your answers to the questions above. You may also include additional comments for the author, including concerns about dual publication, research ethics, or publication ethics. (Please upload your review as an attachment if it exceeds 20,000 characters)

Reviewer #1: Referee Report

• Abstract section is very weak and this section should be more specific.

• Why B3LYP/SVP can you explain?

• Introduction section, FMO, MEP, ADMET, drug-likeness of the title molecule should be expanded with new references such as

1- Synthesis, spectroscopic characterization, DFT, molecular docking and in vitro antibacterial potential of novel quinoline derivatives, Journal of Molecular Structure, Volume 1246, 15 December 2021, 131217

2- Quantum computational, Spectroscopic Investigations on N-(2-((2-chloro-4,5-dicyanophenyl)amino)ethyl)-4-methylbenzenesulfonamide by DFT/TD-DFT with Different Solvents, Molecular Docking and Drug-Likeness Researches, Colloids and Surfaces A Physicochemical and Engineering Aspects 638:128311

3- New Heterocyclic Compound as Carbon Steel Corrosion Inhibitor in 1 M H2SO4, High Efficiency at Low Concentration: Experimental and Theoretical Studies https://doi.org/10.1080/01694243.2022.2034588

• The resolutions of the all figures should be increased.

• I can not see the label of Fig. 1 should be corrected.

• Figure 2 should be transparent form and the labels of atoms should be added.

• Figure 3 should be revised.

• Figure 4-9 should be revised very complex.

• The resolutions of the figures should be increased.

• Why these receptors were select for the molecular docking should be clarified.

• Why both Autodock and MOE are needed for calculations?

• If possible experimental activity should be added.

• Conclusion section should be revised.

MINOR REVISION

Reviewer #2: The paper is written in a style that is not typical of well conceived scientific literature. The main drawback of the paper is that in general there are many values calculated, but no discussion of what they mean and how they compare to other materials. Therefore the reader must find a meaning by him/herself of the computed quantities. On the other hand, many times there are explanations about the physical and chemical meanings of some quantities, which are well known in the literature and should not be explained again. A deep revision of the style of the paper is needed.

Some required minor changes are reported in the following:

- Revise the first sentence of the introduction

- Molecular Dynamics simulations: "For 100 nanoseconds, MD simulations were carried out for both the complexes by using the same

protocol as reported earlier". Add a reference

- remove the word "showing" from the legends of the figures

- revise the first sentence after the caption of Figure 1

- In the sentence just before Table 1, which symmetry constraints are used?

- Table 1 seems to indicate that calculations in the solvent phase are performed in water, but the fourth sentence after Table 1 seems to indicate they are performed in methanol.

- Frontier molecular orbitals: "In gas phase, both the compounds Quinidine and Quinine had shown the same energy gap of 0.15 eV. On contrast in solvent phase (water), again both the compounds had shown the same energy gap of 0.15 eV." There is no contrast.

- Third line before Molecular electrostatic potential: was -> were

- MD simulations Line 9: "the protein's structural structure" revise

- rephrase the part "It is critical for your simulation to achieve a point of convergence, as well as for your residual standard deviation (RMSD) to settle at a constant level. It's likely that your system hasn't achieved equilibrium yet, and that your simulation hasn't run for long enough to offer a thorough assessment of the protein's relative molecular weight distribution"

- "Between 50 and 60 Angstroms, its root mean square deviation was slightly larger". Are the authors sure about the units?"

- Revise the first sentence of conclusions

- Rephrase the sentences "To be truthful, it is not viable to repeat computations for the same ligands because the software is quite lengthy. That is why we calculated the RMSD value using the best poses. Additionally, to validate and verify our findings, co-crystal ligand (NAP) was docked alongside ligands."

- The conclusions are not conclusions in the sense that they do not show the main results of the study.

- Quinidine and quinine in Figure 1 look identical. Please, point out the difference.

- Figure S3: labels and numbers superimpose

- Figure S4: label, numbers and plots superimpose

Reviewer #3: This article contains information and investigation of Quinine and Quinidine as potential Inhibitors of AKR1B1 and AKR1B10. It is not determined inconsistency for dual publication, research ethics, and publication ethics. I think that this manuscript is appropriate to publish in the PLOS ONE.

Reviewer #4: Dear Authors,

I have reviewed your manuscript, and I am expressing my positive feedback. Your study is interesting for the readers of the PLOS One journal, and the obtained results are promising. However, there is space for improvements, so I am requesting revisions according to the following comments:

• You should elaborate why did you use SVP basis set, when the def2-TZVP is mostly recommended? Your molecular are not large and the def2-TZVP basis set should have been used.

• Why the acronym of density functional theory is “DFTs”?

• In the chapter dealing with the global reactivity properties, you have only provided numerical values of the quantities, without proper scientific discussion. Also, that chapter doesn’t contain equations how these descriptors have been calculated.

• Please elaborate why you have performed calculations in both gas and solvent phases?

• Regarding your calculations, also please insert the information about the convergence criteria for SCF and optimization procedures. If you used the default settings, it is enough to mention that the default settings have been used, but in that case please mention which revision of the Gaussian program was used for calculations.

Once you address all of the above-mentioned comments, I will gladly review your manuscript again.

Best regards

**********

6. PLOS authors have the option to publish the peer review history of their article (what does this mean?). If published, this will include your full peer review and any attached files.

If you choose “no”, your identity will remain anonymous but your review may still be made public.

**Do you want your identity to be public for this peer review?** For information about this choice, including consent withdrawal, please see our Privacy Policy.

Reviewer #1: **Yes: **Yusuf Sert

Reviewer #2: No

Reviewer #3: No

Reviewer #4: No

[NOTE: If reviewer comments were submitted as an attachment file, they will be attached to this email and accessible via the submission site. Please log into your account, locate the manuscript record, and check for the action link "View Attachments". If this link does not appear, there are no attachment files.]

While revising your submission, please upload your figure files to the Preflight Analysis and Conversion Engine (PACE) digital diagnostic tool, https://pacev2.apexcovantage.com/. PACE helps ensure that figures meet PLOS requirements. To use PACE, you must first register as a user. Registration is free. Then, login and navigate to the UPLOAD tab, where you will find detailed instructions on how to use the tool. If you encounter any issues or have any questions when using PACE, please email PLOS at figures@plos.org. Please note that Supporting Information files do not need this step.

# Author comment: Author response to Decision Letter 0

**DOI:** 10.1371/journal.pone.0271602.r002

16 May 2022

Reviewer #1: Referee Report

• Abstract section is very weak and this section should be more specific.

Response: The abstract has been revised, as suggested

• Why B3LYP/SVP can you explain?

Response: B3lyp is a functional, which includes exact exchange and GGA corrections in addition to LDA electron-electron and electron-nuclei energy. The weights of the parts were fit to reproduce geometry of a test suite of small molecules. As such use of b3lyp for calculations with heavier atoms is questionable. B3LYP is generally faster than most Post Hartree-Fock techniques and usually yields comparable results. It is also fairly robust for a DFT method. On a more fundamental level, it is not as heavily parameterized as other hybrid functionals, having only 3 where as some have up to 26. Becke's original paper is one of the most cited papers (I think it's number 8) of all time, so B3LYP is well established in the literature and people are less likely to complain about your choice versus a newer functional.

• Introduction section, FMO, MEP, ADMET, drug-likeness of the title molecule should be expanded with new references such as

1- Synthesis, spectroscopic characterization, DFT, molecular docking and in vitro antibacterial potential of novel quinoline derivatives, Journal of Molecular Structure, Volume 1246, 15 December 2021, 131217

2- Quantum computational, Spectroscopic Investigations on N-(2-((2-chloro-4,5-dicyanophenyl)amino)ethyl)-4-methylbenzenesulfonamide by DFT/TD-DFT with Different Solvents, Molecular Docking and Drug-Likeness Researches, Colloids and Surfaces A Physicochemical and Engineering Aspects 638:128311

3- New Heterocyclic Compound as Carbon Steel Corrosion Inhibitor in 1 M H2SO4, High Efficiency at Low Concentration: Experimental and Theoretical Studies https://doi.org/10.1080/01694243.2022.2034588

Response: These references have been incorporated in the revised manuscript

• The resolutions of the all figures should be increased.

Response: The resolution of all figures has been increased, as suggested

• I can not see the label of Fig. 1 should be corrected.

Response: Figure 1 has been revised

• Figure 2 should be transparent form and the labels of atoms should be added.

Response: Figure 2 has been revised

• Figure 3 should be revised.

Response: Figure 3 has been revised

• Figure 4-9 should be revised very complex.

Response: Figures have been revised

• The resolutions of the figures should be increased.

Response: The resolution of all figures has been increased, as suggested

• Why these receptors were select for the molecular docking should be clarified.

Response: As our work aims to identify potent aldo-keto reductase inhibitors that would help to treat the colon cancer, in which the higher levels of AKR1B1 is observed [1]. AKR1B10 was selected just to confirm whether the inhibitor is selective for AKR1B1 or not.

Reference: [1] Taskoparan B, Seza EG, Demirkol S, Tuncer S, Stefek M, Gure AO, Banerjee S, Opposing roles of the aldo-keto reductases AKR1B1 and AKR1B10 in colorectal cancer. Cellular Oncology, 2017:40(6):563-578.

• Why both Autodock and MOE are needed for calculations?

Response: To check the reliability of both softwares. Thus, AutoDock was proved to be the best among the two softwares because it showed less dispersion as compared to MOE on repeated docking protocols.

• If possible experimental activity should be added.

Response: Yes the reviewer is right that computational work required experimental evidence. In our case based on available resources, we have provided the extensive computational work.We have already planned for our upcoming publication and both enzymes are already in purchase demand. All the authors are thankful to the reviewer in advance for considering this study based on computational data. Hope the reviewer will understand our situation.

• Conclusion section should be revised.

Response: Conclusion has been revised

MINOR REVISION

Reviewer #2: The paper is written in a style that is not typical of well conceived scientific literature. The main drawback of the paper is that in general there are many values calculated, but no discussion of what they mean and how they compare to other materials. Therefore the reader must find a meaning by him/herself of the computed quantities. On the other hand, many times there are explanations about the physical and chemical meanings of some quantities, which are well known in the literature and should not be explained again. A deep revision of the style of the paper is needed.

Some required minor changes are reported in the following:

- Revise the first sentence of the introduction

Response: This sentence has been revised

- Molecular Dynamics simulations: "For 100 nanoseconds, MD simulations were carried out for both the complexes by using the same protocol as reported earlier". Add a reference

Response: Thank You for suggesting, the reference was already added but not cited properly. Now it has been corrected

- remove the word "showing" from the legends of the figures

Response: Corrected as suggested

- revise the first sentence after the caption of Figure 1

Response: This sentence has been revised

- In the sentence just before Table 1, which symmetry constraints are used?

Response: It was a typographical mistake, no symmetry constraints were applied

- Table 1 seems to indicate that calculations in the solvent phase are performed in water, but the fourth sentence after Table 1 seems to indicate they are performed in methanol.

Response: It was a typographical mistake which is corrected in revised manuscript. Water was used as a solvent

- Frontier molecular orbitals: "In gas phase, both the compounds Quinidine and Quinine had shown the same energy gap of 0.15 eV. On contrast in solvent phase (water), again both the compounds had shown the same energy gap of 0.15 eV." There is no contrast.

Response: It was a typographical mistake and has been corrected in revised version

- Third line before Molecular electrostatic potential: was -> were

Response: Corrected as suggested

- MD simulations Line 9: "the protein's structural structure" revise

Response: Corrected

- rephrase the part "It is critical for your simulation to achieve a point of convergence, as well as for your residual standard deviation (RMSD) to settle at a constant level. It's likely that your system hasn't achieved equilibrium yet, and that your simulation hasn't run for long enough to offer a thorough assessment of the protein's relative molecular weight distribution"

Response: Suggested part has been rephrased in the revised manuscript

- "Between 50 and 60 Angstroms, its root mean square deviation was slightly larger". Are the authors sure about the units?"

Response: You pointed out right, it is a typo mistake because MD trajectory showed slightly higher RMSD pattern during 50 to 60 nanoseconds of simulation time but mistakenly it was written as 50-60 angstrom.

- Revise the first sentence of conclusions

Response: This has been revised

- Rephrase the sentences "To be truthful, it is not viable to repeat computations for the same ligands because the software is quite lengthy. That is why we calculated the RMSD value using the best poses. Additionally, to validate and verify our findings, co-crystal ligand (NAP) was docked alongside ligands."

Response: It has been rephrased in revised manuscript

- The conclusions are not conclusions in the sense that they do not show the main results of the study.

Response: The conclusion has been revised, as suggested

- Quinidine and quinine in Figure 1 look identical. Please, point out the difference.

Response: You are right, they look alike in optimized structures because they are isomers.

- Figure S3: labels and numbers superimpose

Response: Figure S3 has been revised

- Figure S4: label, numbers and plots superimpose

Response: Figure S4 has been revised

Reviewer #3: 

This article contains information and investigation of Quinine and Quinidine as potential Inhibitors of AKR1B1 and AKR1B10. It is not determined inconsistency for dual publication, research ethics, and publication ethics. I think that this manuscript is appropriate to publish in the PLOS ONE.

Reviewer #4: Dear Authors,

I have reviewed your manuscript, and I am expressing my positive feedback. Your study is interesting for the readers of the PLOS One journal, and the obtained results are promising. However, there is space for improvements, so I am requesting revisions according to the following comments:

• You should elaborate why did you use SVP basis set, when the def2-TZVP is mostly recommended? Your molecular are not large and the def2-TZVP basis set should have been used.

Response: You are correct, def2-TZVP is the most valuable functional but we donot have enough computational resources to test this. For saving the time and computational cost we recommended small SVP basis set. 

• Why the acronym of density functional theory is “DFTs”?

Response: This is a mistake; the correct acronym is DFT and replaced/corrected in the updated paper. 

• In the chapter dealing with the global reactivity properties, you have only provided numerical values of the quantities, without proper scientific discussion. Also, that chapter doesn’t contain equations how these descriptors have been calculated.

Response: Thank you for highlighting this part. We have added the details for these parameters in the revised manuscript. 

• Please elaborate why you have performed calculations in both gas and solvent phases?

Response: The minima corresponding to the gas-phase and solution (PCM model) are definitely not the same. If you're a purist, you should redo all the calculations using the PCM model to get new minima (checked by frequency calculations) and treat the gas-phase calculations just as a good starting point for PCM ones. Although structural differences are typically very small, they will have an effect on energies. The investigated compounds are biological compounds that mostly work according to the body's environment. Usually energy of stabilization/equilibrium change by using the gas phase and solution phase. So, we used both the gas phase and solvent environment so that we know about these parameters. 

• Regarding your calculations, also please insert the information about the convergence criteria for SCF and optimization procedures. If you used the default settings, it is enough to mention that the default settings have been used, but in that case please mention which revision of the Gaussian program was used for calculations.

Response: The Gaussian 09 package (Rev.E.01) with default settings was used for all calculations.

Attachment Submitted filename: Reviewer report.doc Click here for additional data file.

# Peer review: Decision Letter 1

**DOI:** 10.1371/journal.pone.0271602.r003

**Authors:** Joazaizulfazli Jamalis

14 Jun 2022

PONE-D-22-10464R1In-silico Investigations of Quinine and Quinidine as potential Inhibitors of AKR1B1 and AKR1B10: Functional and Structural CharacterizationPLOS ONE

Dear Dr. Ejaz,

Thank you for submitting your manuscript to PLOS ONE. After careful consideration, we feel that it has merit but does not fully meet PLOS ONE’s publication criteria as it currently stands. Therefore, we invite you to submit a revised version of the manuscript that addresses the points raised during the review process.

Please submit your revised manuscript by Jul 29 2022 11:59PM. If you will need more time than this to complete your revisions, please reply to this message or contact the journal office at plosone@plos.org. When you're ready to submit your revision, log on to https://www.editorialmanager.com/pone/ and select the 'Submissions Needing Revision' folder to locate your manuscript file.

Please include the following items when submitting your revised manuscript:

- A rebuttal letter that responds to each point raised by the academic editor and reviewer(s). You should upload this letter as a separate file labeled 'Response to Reviewers'.
- A marked-up copy of your manuscript that highlights changes made to the original version. You should upload this as a separate file labeled 'Revised Manuscript with Track Changes'.
- An unmarked version of your revised paper without tracked changes. You should upload this as a separate file labeled 'Manuscript'.

If you would like to make changes to your financial disclosure, please include your updated statement in your cover letter. Guidelines for resubmitting your figure files are available below the reviewer comments at the end of this letter.

If applicable, we recommend that you deposit your laboratory protocols in protocols.io to enhance the reproducibility of your results. Protocols.io assigns your protocol its own identifier (DOI) so that it can be cited independently in the future. For instructions see: https://journals.plos.org/plosone/s/submission-guidelines#loc-laboratory-protocols. Additionally, PLOS ONE offers an option for publishing peer-reviewed Lab Protocol articles, which describe protocols hosted on protocols.io. Read more information on sharing protocols at https://plos.org/protocols?utm_medium=editorial-email&utm_source=authorletters&utm_campaign=protocols.

We look forward to receiving your revised manuscript.

Kind regards,

Joazaizulfazli Jamalis

Academic Editor

PLOS ONE

Journal Requirements:

Please review your reference list to ensure that it is complete and correct. If you have cited papers that have been retracted, please include the rationale for doing so in the manuscript text, or remove these references and replace them with relevant current references. Any changes to the reference list should be mentioned in the rebuttal letter that accompanies your revised manuscript. If you need to cite a retracted article, indicate the article’s retracted status in the References list and also include a citation and full reference for the retraction notice.

[Note: HTML markup is below. Please do not edit.]

Reviewers' comments:

Reviewer's Responses to Questions

**Comments to the Author**

1. If the authors have adequately addressed your comments raised in a previous round of review and you feel that this manuscript is now acceptable for publication, you may indicate that here to bypass the “Comments to the Author” section, enter your conflict of interest statement in the “Confidential to Editor” section, and submit your "Accept" recommendation.

Reviewer #5: All comments have been addressed

Reviewer #6: (No Response)

Reviewer #7: (No Response)

**********

2. Is the manuscript technically sound, and do the data support the conclusions?

The manuscript must describe a technically sound piece of scientific research with data that supports the conclusions. Experiments must have been conducted rigorously, with appropriate controls, replication, and sample sizes. The conclusions must be drawn appropriately based on the data presented. 

Reviewer #5: Yes

Reviewer #6: Partly

Reviewer #7: Yes

**********

3. Has the statistical analysis been performed appropriately and rigorously? 

Reviewer #5: N/A

Reviewer #6: N/A

Reviewer #7: Yes

**********

4. Have the authors made all data underlying the findings in their manuscript fully available?

The PLOS Data policy requires authors to make all data underlying the findings described in their manuscript fully available without restriction, with rare exception (please refer to the Data Availability Statement in the manuscript PDF file). The data should be provided as part of the manuscript or its supporting information, or deposited to a public repository. For example, in addition to summary statistics, the data points behind means, medians and variance measures should be available. If there are restrictions on publicly sharing data—e.g. participant privacy or use of data from a third party—those must be specified.

Reviewer #5: Yes

Reviewer #6: Yes

Reviewer #7: Yes

**********

5. Is the manuscript presented in an intelligible fashion and written in standard English?

PLOS ONE does not copyedit accepted manuscripts, so the language in submitted articles must be clear, correct, and unambiguous. Any typographical or grammatical errors should be corrected at revision, so please note any specific errors here.

Reviewer #5: Yes

Reviewer #6: No

Reviewer #7: No

**********

6. Review Comments to the Author

Please use the space provided to explain your answers to the questions above. You may also include additional comments for the author, including concerns about dual publication, research ethics, or publication ethics. (Please upload your review as an attachment if it exceeds 20,000 characters)

Reviewer #5: The manuscript is concise, informative, and well written. The authors have thoroughly shed light on the Quinine and Quinidine as potential inhibitors of AKR1B1 and AKR1B10. All the suggestions of the reviewers are appropriately incorporated. Findings of the study favour Quinidine as a better inhibitor of AKR1B1 and Quinine as a plausible inhibitor of AKR1B10.The article is helpful in the concerned field of pharmacopoeia and rational drug design. Therefore, it is recommendable for publication in the journal. However, authors must check once again for typos and grammar mistakes throughout the manuscript.

Reviewer #6: The reviewer comments are attached . Author needs to work on the pointed areas by giving suitable justifications and explanations in the manuscript.

Reviewer #7: Dear editor,

Have a nice day. The authors carried out some In-silico tests for Quinine and Quinidine as potential Inhibitors of AKR1B1 and AKR1B10.

The manuscript lacks novelty in some points

1- That the bases to select Quinine and Quinidine as a potential target for AKR1B1 and AKR1B10? Are these compounds have some sort of similarity with the previously reported AKR1B1 and AKR1B10 inhibitors?? If this is the case, confirm the structural similarity.

2- The binding modes of Quinine and Quinidine need more clarification. Compare the binding mode of Quinine and Quinidine with that of co-crystallized ligand.

3- In vitro cytotoxicity of Quinine and Quinidine against cancer cell line is necessary.

4- In vitro enzymatic inhibition is necessary.

**********

7. PLOS authors have the option to publish the peer review history of their article (what does this mean?). If published, this will include your full peer review and any attached files.

If you choose “no”, your identity will remain anonymous but your review may still be made public.

**Do you want your identity to be public for this peer review?** For information about this choice, including consent withdrawal, please see our Privacy Policy.

Reviewer #5: **Yes: **Dr. Mohammad Kalim Ahmad Khan

Reviewer #6: No

Reviewer #7: No

**********

[NOTE: If reviewer comments were submitted as an attachment file, they will be attached to this email and accessible via the submission site. Please log into your account, locate the manuscript record, and check for the action link "View Attachments". If this link does not appear, there are no attachment files.]

While revising your submission, please upload your figure files to the Preflight Analysis and Conversion Engine (PACE) digital diagnostic tool, https://pacev2.apexcovantage.com/. PACE helps ensure that figures meet PLOS requirements. To use PACE, you must first register as a user. Registration is free. Then, login and navigate to the UPLOAD tab, where you will find detailed instructions on how to use the tool. If you encounter any issues or have any questions when using PACE, please email PLOS at figures@plos.org. Please note that Supporting Information files do not need this step.

Attachment Submitted filename: PONE-Reviewer Comments-7th June 2022.docx Click here for additional data file.

# Author comment: Author response to Decision Letter 1

**DOI:** 10.1371/journal.pone.0271602.r004

27 Jun 2022

Reviewer #5: The manuscript is concise, informative, and well written. The authors have thoroughly shed light on the Quinine and Quinidine as potential inhibitors of AKR1B1 and AKR1B10. All the suggestions of the reviewers are appropriately incorporated. Findings of the study favour Quinidine as a better inhibitor of AKR1B1 and Quinine as a plausible inhibitor of AKR1B10.The article is helpful in the concerned field of pharmacopoeia and rational drug design. Therefore, it is recommendable for publication in the journal. However, authors must check once again for typos and grammar mistakes throughout the manuscript.

Response: First of all Thanks to the reviewer for considering the research article. Now all the typos and grammar mistakes have been corrected in the revised manuscript

Reviewer #6: The reviewer comments are attached. Author needs to work on the pointed areas by giving suitable justifications and explanations in the manuscript.

1. The abstract has been revised well. The very first line does not convey the message. Rephrase. 

Response: The abstract has been revised as suggested

2. In results and discussion section, the author has given the following paragraph: In gas phase, both the compounds Quinidine and Quinine had shown the same energy gap of 0.15 eV…………. . But have not fully justified the meaning of values or their impact on the reactivity of compounds as inhibitors for the targeted protein. Moreover, I do not find enough explanation in terms of DFT study which indeed is quite powerful tool for giving reliable predictions about structure reactivity pattern besides many other chemical features. In a nutshell, this section seems more like a general theory for DFT rather than case study details and explanations. There is a lot room for improvement in this study. 

Response: The required justifications and corrections have be incorporated in the revised manuscript as suggested. 

3. The manuscript needs revision in terms of English language and grammar like e.g. In results and discussion section: “Here, is the case with Quinidine and Quinine shows the same significant values as they are the optical isomers of each other.” 

& 

In introduction section: “From this study, may the quinolone derivatives be proved lead compounds for the treatment of colon cancer by inhibiting aldo-keto reductase (17).”

Response: All the grammatical errors have been removed from the revised manuscript

4. There is a lack in connection between the generalization for Global chemical reactivity descriptors values and the values obtained for the test molecules with ultimate effect on reactivity with the targeted proteins. Moreover, authors would have considered predicting the SAR for the inhibitor molecules based on different parameters studied by DFT. A lot much can be improved in DFT section. It seems authors are unable to fully explain/justify the results systematically besides having calculation of values for various useful parameters. 

Response: It has been revised, as suggested.

5. Authors state in molecular docking section as:” The hydrogen bonding is significant in protein ligand interaction to check the inhibitory action.” That is true but not a universal rule indeed for docked ligands. To rationalize this statement authors, need to justify in their case by giving suitable explanation in terms of structure activity relationship. 

Response: The structure activity relationship has been incorporated in the revised manuscript as suggested. 

6. Incomplete explanation of the following statement: “LogP is used to determine a compound's hydrophilicity; if the LogP value is negative, the compound is hydrophilic. Compounds are lipophilic.” Which value type indicates lipophilic behavior, not explained. 

Response: It has been explained in the revised manuscript

Reviewer #7: Dear editor,

Have a nice day. The authors carried out some In-silico tests for Quinine and Quinidine as potential Inhibitors of AKR1B1 and AKR1B10.

The manuscript lacks novelty in some points

1- That the bases to select Quinine and Quinidine as a potential target for AKR1B1 and AKR1B10? Are these compounds have some sort of similarity with the previously reported AKR1B1 and AKR1B10 inhibitors?? If this is the case, confirm the structural similarity.

Response: The detailed rationale has been incorporated in the revised manuscript as suggested

2- The binding modes of Quinine and Quinidine need more clarification. Compare the binding mode of Quinine and Quinidine with that of co-crystallized ligand.

Response: The suggestion has been addressed in the revised manuscript

3- In vitro cytotoxicity of Quinine and Quinidine against cancer cell line is necessary.

Response: Yes the reviewer is right that computational work required experimental evidence. Our studies are based on available resources. We have put the purchase demand of targeted enzymes and cell lines, and we will consider it in our upcoming publications. Hope the reviewer will understand our situation and accept this appology. However, a detailed insilico studies against cancer markers have been added to support our results. 

4- In vitro enzymatic inhibition is necessary.

Response: The reviewer gave a very fruitful suggestion and we agree that without in-vitro justification out results are partially validated. We have provided more in-silico justifications that our compounds have strong anticancer potential. We have put purchase demand of our targeted Enzymes and they are in process. This will take time and at this stage, authors of this study are thankful in advance to the reviewer to consider our justifications of this point. We are working on more compounds and we will incorporate in-vitro studies in our upcoming publication along with the justification of current compounds.

Attachment Submitted filename: PONE-Reviewer Comments-7th June 2022.docx Click here for additional data file.

# Editor report: Decision Letter 2

**DOI:** 10.1371/journal.pone.0271602.r005

**Authors:** Joazaizulfazli Jamalis

4 Jul 2022

In-silico Investigations of Quinine and Quinidine as potential Inhibitors of AKR1B1 and AKR1B10: Functional and Structural Characterization

PONE-D-22-10464R2

Dear Dr. Ejaz,

We’re pleased to inform you that your manuscript has been judged scientifically suitable for publication and will be formally accepted for publication once it meets all outstanding technical requirements.

Within one week, you’ll receive an e-mail detailing the required amendments. When these have been addressed, you’ll receive a formal acceptance letter and your manuscript will be scheduled for publication.

An invoice for payment will follow shortly after the formal acceptance. To ensure an efficient process, please log into Editorial Manager at http://www.editorialmanager.com/pone/, click the 'Update My Information' link at the top of the page, and double check that your user information is up-to-date. If you have any billing related questions, please contact our Author Billing department directly at authorbilling@plos.org.

If your institution or institutions have a press office, please notify them about your upcoming paper to help maximize its impact. If they’ll be preparing press materials, please inform our press team as soon as possible -- no later than 48 hours after receiving the formal acceptance. Your manuscript will remain under strict press embargo until 2 pm Eastern Time on the date of publication. For more information, please contact onepress@plos.org.

Kind regards,

Joazaizulfazli Jamalis

Academic Editor

PLOS ONE

Additional Editor Comments (optional):

Reviewers' comments:

# Editor report: Acceptance letter

**DOI:** 10.1371/journal.pone.0271602.r006

**Authors:** Joazaizulfazli Jamalis

7 Oct 2022

PONE-D-22-10464R2 

In-silico Investigations of Quinine and Quinidine as potential Inhibitors of AKR1B1 and AKR1B10: Functional and Structural Characterization 

Dear Dr. Ejaz:

I'm pleased to inform you that your manuscript has been deemed suitable for publication in PLOS ONE. Congratulations! Your manuscript is now with our production department. 

If your institution or institutions have a press office, please let them know about your upcoming paper now to help maximize its impact. If they'll be preparing press materials, please inform our press team within the next 48 hours. Your manuscript will remain under strict press embargo until 2 pm Eastern Time on the date of publication. For more information please contact onepress@plos.org.

If we can help with anything else, please email us at plosone@plos.org. 

Thank you for submitting your work to PLOS ONE and supporting open access. 

Kind regards, 

PLOS ONE Editorial Office Staff

on behalf of

Dr. Joazaizulfazli Jamalis 

Academic Editor

PLOS ONE
